# An Internet-Based Cognitive Behavioral Therapy Program Adapted to Patients With Cardiovascular Disease and Depression: Randomized Controlled Trial

**DOI:** 10.2196/14648

**Published:** 2019-10-03

**Authors:** Peter Johansson, Mats Westas, Gerhard Andersson, Urban Alehagen, Anders Broström, Tiny Jaarsma, Ghassan Mourad, Johan Lundgren

**Affiliations:** 1 Department of Social and Welfare Studies Linköping University Norrköping Sweden; 2 Department of Internal Medicine Department of Medical Health Sciences Linköping University Norrköping Sweden; 3 Department of Behavioural Sciences and Learning Linköping University Linköping Sweden; 4 Department of Clinical Neuroscience Karolinska Institutet Stockholm Sweden; 5 Department of Medical Health Sciences Linköping University Linköping Sweden; 6 Department of Nursing Jönköping University Jönköping Sweden

**Keywords:** cardiovascular disease, depression, cognitive behavior therapy, internet, randomized controlled trial

## Abstract

**Background:**

Depression is a common cause of reduced well-being and prognosis in patients with cardiovascular disease (CVD). However, there is a lack of effective intervention strategies targeting depression.

**Objective:**

The study aimed to evaluate the effects of a nurse-delivered and adapted internet-based cognitive behavioral therapy (iCBT) program aimed at reducing depression in patients with CVD.

**Methods:**

A randomized controlled trial was conducted. A total of 144 patients with CVD with at least mild depression (Patient Health Questionnaire–9 [PHQ-9] score ≥5) were randomized 1:1 to a 9-week program of iCBT (n=72) or an active control participating in a Web-based discussion forum (online discussion forum [ODF], n=72). The iCBT program, which included 7 modules, was adapted to fit patients with CVD. Nurses with an experience of CVD care provided feedback and a short introduction to cognitive behavioral therapy. The primary outcome, depression, was measured using PHQ-9. Secondary outcomes were depression measured using the Montgomery-Åsberg Depression Rating Scale–self-rating version (MADRS-S), health-related quality of life (HRQoL) measured using Short Form 12 (SF-12) survey and EuroQol Visual Analogue Scale (EQ-VAS), and the level of adherence. An intention-to-treat analysis with multiple imputations was used. Between-group differences in the primary and secondary outcomes were determined by the analysis of covariance, and a sensitivity analysis was performed using mixed models.

**Results:**

Compared with ODF, iCBT had a significant and moderate treatment effect on the primary outcome depression (ie, PHQ-9; mean group difference=−2.34 [95% CI −3.58 to −1.10], *P*<.001, Cohen *d*=0.62). In the secondary outcomes, compared with ODF, iCBT had a significant and large effect on depression (ie, MADRS-S; *P*<.001, Cohen *d*=0.86) and a significant and moderate effect on the mental component scale of the SF-12 (*P*<.001, Cohen *d*=0.66) and the EQ-VAS (*P*<.001, Cohen *d*=0.62). Overall, 60% (n=43) of the iCBT group completed all 7 modules, whereas 82% (n=59) completed at least half of the modules. No patients were discontinued from the study owing to a high risk of suicide or deterioration in depression.

**Conclusions:**

Nurse-delivered iCBT can reduce depression and improve HRQoL in patients with CVD, enabling treatment for depression in their own homes and at their preferred time.

**Trial Registration:**

ClinicalTrials.gov NCT02778074; https://clinicaltrials.gov/ct2/show/NCT02778074

## Introduction

### Background

Depression is highly prevalent in patients with cardiovascular disease (CVD; ie, atrial fibrillation or atrial flutter, ischemic heart disease, and heart failure) [1], with an estimated prevalence of 20% to 40%. Patients with CVD with depression experience reduced health-related quality of life (HRQoL) and have an increased risk of cardiovascular complications and premature death [1]. Potential bio-behavioral mechanisms underlying the negative effects of depression include impairment of self-care activities and/or elevations in the stress and inflammatory response systems [1,2]. This underscores the importance of treating depression in CVD. Pharmacological treatment of depression may be an option in patients with CVD, but the effects are small [1]. Moreover, such treatment poses a challenge as adding another medication to the existing complex medical treatment may be perceived as burdensome and might increase the risk of developing side effects [3].

### Psychological Interventions for Depression in Cardiovascular Disease

One possible alternative and complementary treatment option is psychological interventions that patients with CVD also seem to prefer to antidepressant treatment [4]. A recent systematic review has reported that psychological interventions in CVD, such as cognitive behavioral therapy (CBT), have small effects on depression in patients with CVD [5]. This, however, does not necessarily mean that CBT is ineffective in CVD. One explanation for the small effects reported may be the heterogeneity of the studies included in the systematic review [5]. Many of them evaluate the complex interventions based on different multiple components, which are often poorly described. Moreover, many of the studies in the review also evaluate non-CBT interventions. It is therefore problematic to determine which types of interventions will work. Another important issue is that many psychological interventional studies of CVD have included samples irrespective of whether the patient has elevated levels of depression or not [5]. This will lead to an increased risk of a floor effect, meaning that participants with no or low levels of depressive symptoms will be unlikely to experience a decrease in depression as a response to the intervention. However, a recent meta-analysis of 12 randomized controlled trials (RCTs) evaluating the effectiveness of CBT on patients with CVD with depression and/or anxiety reported significantly lower depression scores at follow-up compared with controls, which in most cases was care as usual [6]. This suggests that interventions based on CBT or CBT principles could be a treatment option for depression in patients with CVD with increased levels of depressive symptoms. However, one barrier to the implementation of CBT in current clinical and cardiac care is low access to psychotherapists, leading to a *treatment-demand gap*. A solution could be internet-based CBT (iCBT), which can be provided at low cost and has been proven effective in patients with depression [7,8]. iCBT can be delivered in a guided format (ie, support and/or encouragement and/or feedback on homework assignments [9]) or unguided; however, guided iCBT tends to be more effective [10]. One important aspect that may facilitate the implementation of guided iCBT in clinical care is that it can be delivered by health care professionals with little or no specific training, without reducing the effect of the treatment [11]. Another advantage of iCBT is that it enables patients with CVD to access CBT in their own homes and at a time that suits them. However, generic iCBT programs may not be optimal for targeting depression in patients with chronic diseases, as these programs are not tailored to the context of the disease [12,13].

### Objectives

In this study, we therefore aim to evaluate the effect of a nurse-delivered, tailored iCBT program designed to reduce depression in patients with CVD.

## Methods

### Study Design and Population

In this RCT, we recruited patients from medical and cardiology clinics at 4 hospitals in southeastern Sweden. Invitations were sent by post to all patients with at least one diagnosis of atrial fibrillation or atrial flutter (International Classification of Diseases [ICD] codes I48 or I49), coronary heart disease (ICD codes I20 or I25), or heart failure (ICD codes I42 or I50) and at least one outpatient visit or hospitalization during the previous year. Recruitment took place in 3 different rounds in January 2017 (hospital 1), October 2017 (hospital 2), and February 2018 (hospitals 3 and 4), and a total of 11,992 patients were approached. Patients interested in participating were instructed to register on the study website (Multimedia Appendix 1), which is a secure website requiring 2-factor authentication to access the study platform [14]. No compensation was provided for participating in the study.

Patients were eligible for inclusion if they were aged 18 years or above and were receiving CVD treatment according to the current guidelines for heart failure, coronary artery disease, and atrial fibrillation from the European Society of Cardiology [15-17], had stable CVD (New York Heart Association [NYHA] class I-III), with no hospitalization related to CVD in the past 4 weeks, and suffered at least mild depressive symptoms (Patient Health Questionnaire–9 [PHQ-9] score ≥5 [18]). Furthermore, patients needed to have regular access to a computer with an internet connection, have access to a mobile phone, and be willing to participate in a treatment program for their depression. The exclusion criteria were severe CVD (ie, NYHA IV) or another comorbid life-threatening disease as assessed by a study nurse, severe depression assessed as requiring acute treatment, and not being willing to dedicate 3 to 4 hours per week to participate in the program.

Patients who had registered on the study website were asked to complete a Web-based screening form that collected data about depression as assessed by PHQ-9, demographics, smoking and alcohol habits, CVD diagnosis, time since diagnosis of CVD, NYHA classification, comorbidities, and medications for CVD, depression, sleep problems, and anxiety. Patients assessed as potential participants (ie, had CVD and scored ≥5 on the PHQ-9, including the presence of at least one of the two core symptoms of depression—depressed mood and/or loss of interest) were contacted by telephone by study nurses, with clinical experience of psychiatric and cardiac care, who gave information about the study, answered questions, and checked any uncertainties about the screening form. They also assessed the severity of CVD and depression. During the telephone interview, the Mini International Neuropsychiatric Interview (MINI) version 5 panel A (ie, depression) and panel C (ie, suicidal ideation) were used to establish the presence of at least mild depression and severity (ie, the presence or absence of core symptoms, depression severity, and suicidal ideation). Those who fulfilled the inclusion criteria and did not exhibit any of the exclusion criteria were included in the study. All included participants signed a paper to give written informed consent. Self-reported data were collected via a set of questionnaires that were completed through the study website. Data were collected at baseline and after study completion at 9 weeks. The study was approved by the regional ethical review board in Linköping, Sweden (number 2016/72-31) and is registered at clinicaltrials.gov (NCT02778074).

### Randomization and Masking

The randomization was performed by a study team member (GM) who was blinded to screening and baseline data. None of the telephone interviewers had access to the random sequence. Patients were randomized 1:1 to the 9-week iCBT program (intervention group) or an online discussion forum (ODF; active control group) generated by an independent statistician using Stata version 13 proc Ralloc (StataCorp LLC) with a block size of 2. Masking of patients was not possible as the intervention is a cognitive behavioral intervention. It was not necessary to mask outcome measures as these were automatically collected via the study platform.

### Procedures

The intervention group participated in a 9-week iCBT program that was initially adapted to fit patients with heart failure and depression and had undergone pilot testing. Its theoretical underpinnings have been described in previous publications [19,20]. The iCBT program, which emphasizes behavioral components, comprises 7 modules: goal setting, psychoeducation, problem solving, behavioral activation part 1 (2 weeks) and part 2 (2 weeks), and a summary module. For this study, the program was further developed to fit a broader group of cardiac patients by adding psychoeducative modules about coronary artery disease, atrial fibrillation, and atrial flutter. Each module comprised a text, short videos, and weekly homework assignments. A table overview of the content of the iCBT program and screenshots of different modules and homework assignments can be found in Multimedia Appendix 2. No changes were made to the program during the trial. Written feedback was provided on each assignment at the end of each week by 3 nurses (PJ, JL, and MW) from the study team, who had experience in cardiology and psychiatry and had taken a short course in iCBT. The nurses participated in a 2-day course about the foundations and uses of iCBT, recent research, practical training in the use of iCBT, and how to give feedback to participants. The nurses were also provided with a handbook about iCBT [21]. The course was run by a well-recognized company specializing in education in psychological treatment. No formal assessment of the nurses’ learning was undertaken. The feedback focused on encouragement, and confirming and reflecting upon the patients’ homework assignments, preparing for the next module [9], and, when required, discussing psychoeducative CVD-related issues (eg, sexuality, treatment side effects, and symptoms). Participants in the iCBT group also had the opportunity to ask questions about the feedback or the content of the module using a message function on the study platform. These questions were answered within 24 hours during working days. The nurses had the opportunity to consult each other, as well as a clinical psychologist (GA) or a cardiologist (UA), if needed. Participants who did not complete the homework assignments received a maximum of 3 written reminders by email during a consecutive period of 2 weeks.

As recommended in a systematic review [13], we used an active control group, who participated in a Web-based moderated discussion forum (ie, ODF group), where new discussion topics were presented each week over a 9-week period. During participation in the ODF, no individual feedback was provided. However, participants could contact the moderator for support regarding how to use the ODF and for technical support. The topic was introduced without any extended background in a presentation comprising statements and questions such as: What are the symptoms of CVD? Do you have any tips you would like to share on how you can handle the symptoms of CVD? How do you think that depressive symptoms and CVD affect the relationship between you and your significant others? Do you have any suggestions about how to handle problems related to feeling depressed or downhearted? The discussion took place in writing. One of the members of the study group (ie, GM) was responsible for monitoring the ODF. The monitoring of the ODF focused on assessing whether there was a good atmosphere between the discussants and to control and correct if a discussant suggested management strategies that could be seen as harmful. After 9 weeks, the participants in the ODF were offered the iCBT program. This information was provided in writing on the study homepage and orally during the telephone interview.

For safety issues, and as requested by the ethical review board, we screened weekly for suicidality and worsening of depressive symptoms using the Montgomery-Åsberg Depression Rating Scale–self-rating Scale (MADRS-S) both in the iCBT and the ODF [22]. Patients who scored 5 or higher on MADRS-S item 9 (zest for life) were contacted by the research team and, if necessary, advised to seek acute psychiatric care.

### Assessments

Participants who did not complete the questionnaires received a maximum of 3 automated reminders.

#### Primary Outcome

PHQ-9 was used to measure the level of depression at baseline and at 9-week follow-up. The instrument comprised 9 items rated on a 4-point scale (not at all, several days, more than half the days, and nearly every day) providing a summary score ranging from 0 to 27 [23]. A score of 0 to 4 indicates no or minimal depressive symptoms, scores of 5 to 9 suggest mild depressive symptoms, scores of 10 to 14 indicate moderate depressive symptoms, and scores >15 indicate severe depressive symptoms. The PHQ-9 has been found to be reliable and valid for detecting depression [18,23] and also in patients with CVD (ie, heart failure) [24]. The PHQ-9 has also been found to be valid in the computerized format [25].

#### Secondary Outcomes

MADRS-S [22] was used as a security measurement for depressive symptoms and suicidal thoughts during the intervention. MADRS-S comprises 9 items rated on a 7-point scale with a maximum score of 54. Higher scores indicate more symptoms of depression. Scores of 0 to 12 on the MADRS-S have been proposed to indicate no depression, scores of 13 to 19 suggest mild depression, and scores of 20 to 54 indicate moderate or severe depression [26]. MADRS-S has been found to be a valid and reliable tool when administered over the internet [27].

HRQoL was measured using the Short Form 12 (SF-12) survey [28] and EuroQol Visual Analogue Scale (EQ-VAS) [29]. The SF-12 measures HRQoL via 12 items selected from the Short Form-36 [28]. Results of the SF-12 are summed into the physical component score (PCS) and the mental component score (MCS). The instrument has been used in a large number of studies and populations, including patients with CVD [30]. The EQ-VAS is a visual analog of HRQoL scale with endpoints labeled “the best health you can imagine,” marked as 100, and “the worst health you can imagine,” marked as 0.

Adherence was determined by the number of completed modules (iCBT group) and the number of activities in the ODF (ODF group), which was provided by the study platform. As recommended, we have also reported the time spent for feedback on homework assignments and responses to messages sent from the participants [13]. This was measured by clocking time from the start to the end of each feedback session or message sent.

### Statistical Analysis

A power calculation based on effect size in the meta-analysis of iCBT [31] showed that a total of 122 participants would be needed to detect at least a moderate effect size on depression (effect size=0.5, alpha=.05 [*z*=1.96], power=0.80 [*z*=0.84]). Owing to expected dropouts, the size of the study sample was set to 140 patients. The characteristics of patients’ baseline data were analyzed using descriptive statistics. Primary and secondary outcome data were checked for normal distribution by visual inspection or Q-Q plots. All variables were found to be suitable for parametric analysis. The primary analysis for comparison between groups (ie, iCBT vs ODF) was made on an intention-to-treat basis. We followed the recommendations of the European Medicines Agency for statistical analysis [32] and used analysis of covariance (ANCOVA), which allows adjusting for baseline scores and regression to the mean [33]. Missing data in the ANCOVA were imputed using last observation carried forward (LOCF) as no consensus on how to best pool *F* statistics was available [34]. However, LOCF has received criticism [35]; therefore, we also applied mixed-models analysis with multiple imputed data as a sensitivity analysis. A total of 40 imputations were performed using the outcome variables and variables from baseline characteristics that had a correlation *r*≥0.5 with the outcome variables as predictors [36]. Multiple imputed datasets, as well as raw data on primary outcome are available in Multimedia Appendix 3. Effect sizes were calculated using Cohen *d*, with a small effect considered to be a value between 0.20 and 0.49, a moderate effect between 0.50 and 0.79, and a value above 0.80 considered to be a large effect. A per-protocol analysis was performed to evaluate categorical improvements in depression as measured by minimal clinically important change, defined by a decrease of 5 points or more on the PHQ-9 [37]; the proportion of nondepressed participants (ie PHQ-9 score <5) at 9-week follow-up; and the proportion of nondepressed or mildly depressed participants (ie, PHQ-9 score <10) at 9-week follow-up. On the basis of these analyses, the numbers needed to treat (NNT) were calculated. We also calculated NNT for MADRS-S as a categorical variable (no depression=MADRS-S score 0-12; depression=MADRS-S score ≥13). A per-protocol analysis was also performed to analyze and compare the change in levels of depression in relation to the number of CBT modules and the number of activities in the ODF completed (ie, adherence to the program). Statistical analysis was performed using IBM SPSS version 23. *P* value <.05 was considered as significant

## Results

### Population

**Figure 1:**
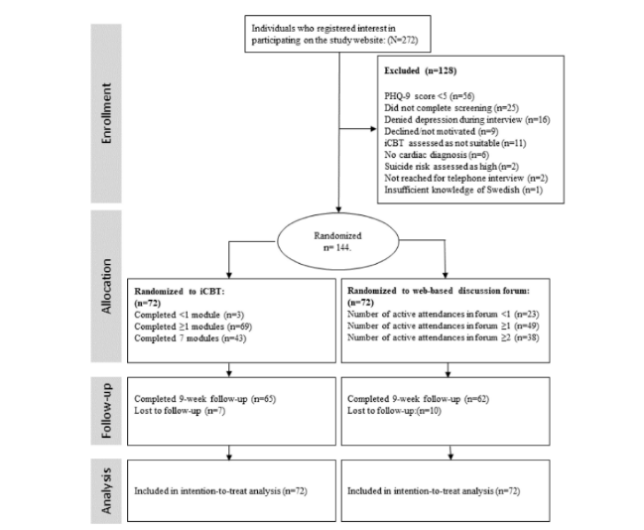
Trial profile. PHQ-9: Patient Health Questionnaire–9; iCBT: internet-based cognitive behavioral therapy.

Out of 11,992 invitations sent to potential participants, 272 (2.26%) registered interest and were screened. Out of these, 144 were included and randomized to either iCBT (n=72) or ODF (n=72). The main reasons for exclusion were a screening score of <5 on PHQ-9 (20%, n=56), not completing the screening form (9%, n=20), or denying depression during the telephone interview (6%, n=16; Figure 1). The number of patients who did not complete the 9-week trial period was similar in the 2 groups (iCBT: 10%, n=7, ODF: 14%, n=10).

Baseline characteristics (Table 1) were similar between the 2 groups. The mean age was 63 (SD 12) years (age span 26-87 years), 38% (n=55) were women and 49% (n=70) had a college or university level of education. With regard to cardiac diagnosis, 56% (n=81) had atrial fibrillation or atrialflutter, 44% (n=65) had coronary heart disease, and 26% (n=38) had heart failure. More than one-quarter of the total population had 2 or more cardiac diagnoses (ie, 28%, n=40). In addition, 53% (n=76) had hypertension, 15% (n=21) had diabetes, and 13% (n=19) had a previous stroke or transitory ischemic attack. With regard to pharmacological treatment for CVD, 76% (n=110) were on beta-blockers, 48% (n=67) were on renin-angiotensin-aldosterone system blocking agents, and 89% (n=110) used antiplatelets or anticoagulants. The mean score on PHQ-9 was 10.47 (SD 4.78) and 14% (n=20) had been prescribed antidepressant treatment.

**Table 1:** Baseline characteristics of participants randomized to internet-based cognitive behavioral therapy or online discussion forum.

Characteristics		Internet-based cognitive behavioral therapy (n=72)	Web-based discussion forum (n=72)
**Sex, n (%)**			
	Male	47 (65)	42 (58)
	Female	25 (35)	30 (42)
Age (years), mean (SD)		61 (13)	64 (12)
**Education, n (%)**			
	Elementary	7 (10)	12 (17)
	Upper secondary/high school	16 (22)	21 (29)
	Postsecondary vocational education	12 (17)	6 (8)
	College/university	37 (51)	33 (46)
**Occupation, n (%)**			
	Working	26 (36)	18 (25)
	Sick leave/disability pension/unemployed	9 (13)	10 (14)
	Retired	32 (44)	36 (50)
	Other	5 (7)	8 (11)
Living alone, n (%)		19 (26)	19 (26)
**Smoking, n (%)**			
	Never	33 (46)	36 (50)
	Ex-smoker	37 (51)	33 (46)
	Smoker	2 (3)	3 (4)
**Alcohol consumption, n (%)**			
	0-4 units per week	51 (71)	58 (80)
	5-9 units per week	17 (24)	10 (14)
	10-14 units per week	3 (4)	4 (6)
	15 or more units per week	1 (1)	0 (0)
**Cardiovascular disease, n (%)**			
	Myocardial infarction/angina	34 (47)	29 (40)
	Atrial fibrillation	40 (56)	41 (57)
	Heart failure	18 (25)	20 (28)
**Number of cardiac diagnoses, n (%)**			
	1	52 (72)	52 (72)
	2	15 (21)	17 (24)
	3	5 (7)	3 (4)
**Time since cardiac diagnosis, n (%)**			
	<6 months	7 (10)	9 (13)
	>6 months	61 (85)	62 (86)
	Do not know	4 (5)	1 (1)
**Comorbidities, n (%)**			
	Hypertension	36 (50)	40 (56)
	Diabetes	8 (11)	13 (18)
	Pulmonary disease	7 (10)	8 (11)
	Stroke and/or TIA^a^	9 (13)	10 (14)
	Renal disease	3 (4)	2 (3)
	Cancer	7 (10)	9 (13)
	Other psychiatric disorder	4 (6)	8 (11)
**NYHA^b^ class, n (%)**			
	1	23 (32)	18 (25)
	2	25 (35)	28 (39)
	3	24 (33)	26 (36)
**Cardiovascular disease medications, n (%)**			
	RAAS blockade^c^	34 (47)	35 (49)
	Beta-blocker	55 (76)	55 (76)
	MRA^d^	5 (7)	6 (8)
	Neprilysin inhibitor	0 (0)	1 (1)
	Antiplatelet/anticoagulants	63 (88)	65 (90)
	Statins	36 (50)	33 (46)
	Diuretics	14 (19)	19 (26)
	Nitroglycerine	15 (21)	15 (21)
	Rhythm stabilizing agents	9 (13)	8 (11)
**Depression medications, n (%)**			
	Antidepressants	7 (10)	13 (18)
	Anxiolytics	7 (10)	12 (17)
	Sleep medication	23 (40)	19 (26)

^a^TIA: transient ischemic attack.

^b^NYHA: New York Heart Association function classification.

^c^RAAS blockade: renin-angiotensin-aldosterone system blocking agents.

^d^MRA: mineral receptor antagonists.

### Outcomes

**Figure 2:**
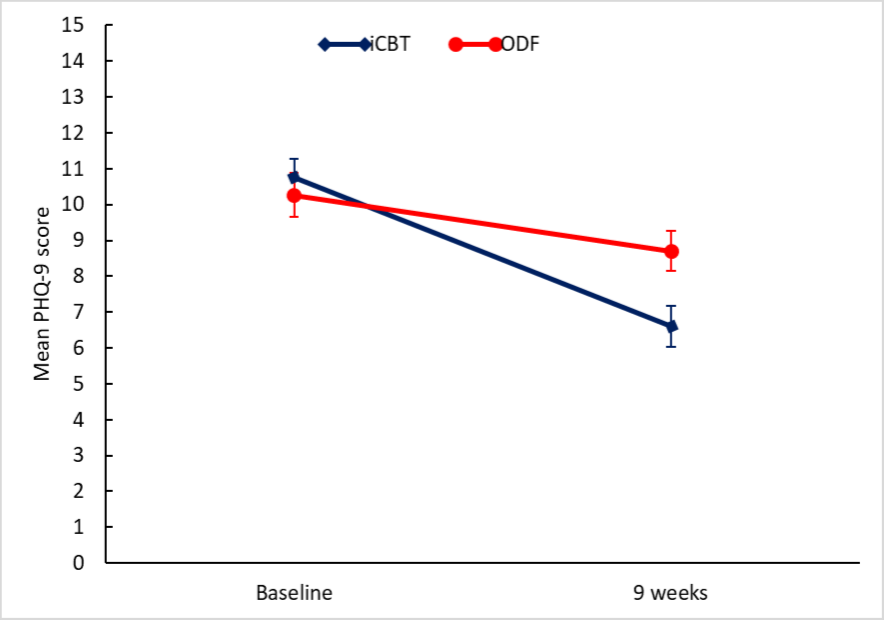
Effects of the internet-based cognitive behavioral therapy (iCBT) program. The figure shows the change in mean scores from baseline to 9-week follow-up as measured by Patient Health Questionnaire–9 (PHQ-9) in those allocated to iCBT (n=72) or Web-based discussion forum (ODF, n=72). Error bars show SE.

The intention-to-treat analysis (iCBT n=72 and ODF n=72) of the primary outcome of depression as measured by PHQ-9 at the 9-week follow-up showed a statistically significant moderate treatment effect of iCBT compared ODF (mean group difference -2.34 [95 % CI -3.58 to -1.10], *P*<.001, Cohen *d*=0.62) (Table 2 and Figure 2). The mixed-model analysis based on multiple imputation data showed similar figures for the primary outcome (mean group difference -2.40 [95% CI −3.93 to −0.87], *P*=.002, Cohen *d*=0.51; Multimedia Appendix 3). In the secondary outcomes, depression measured by MADRS-S, iCBT had a significant and large effect (*P*<.001, Cohen *d*=0.86) compared with ODF. We also found significant improvements and moderate effects owing to iCBT on EQ-VAS (*P*<.001, Cohen *d*=0.62) and on the MCS of the SF-12 (*P*<.001, Cohen *d*=0.66). In the PCS of the SF-12, a small but nonsignificant improvement owing to iCBT was found (*P*=.06, Cohen *d*=0.32; Table 2). The mixed-model analysis for the secondary outcomes showed similar figures and are reported in Multimedia Appendix 3.

**Table 2:** Treatment effects for the primary and secondary outcomes. All data are imputed using last observation carried forward.

Questionnaires	Internet-based cognitive behavioral therapy (n=72), mean (SD)		Online discussion forum (n=72), mean (SD)		Mean between-group treatment difference (95% CI)	*P* value	Effect size (*d*)
	Baseline	9 weeks	Baseline	9 weeks			
PHQ-9^a^	10.71 (4.47)	6.63 (4.76)	10.22 (5.10)	8.68 (4.60)	−2.34 (−3.58 to −1.10)	<.001	0.62
MADRS-S^b^	18.27 (6.98)	10.90 (7.45)	17.67 (6.19)	16.10 (7.93)	−5.58 (−7.72 to −3.44)	<.001	0.86
EQ-VAS^c^	53.31 (20.03)	65.11 (21.81)	57.15 (18.10)	56.99 (22.08)	10.83 (5.02 to 16.64)	<.001	0.62
PCS12^d^	39.70 (10.07)	41.84 (10.56)	37.63 (10.98)	37.80 (11.61)	2.46 (−0.11 to 5.03)	.06	0.32
MCS12^e^	35.88 (9.22)	43.41 (11.04)	36.38 (10.02)	38.03 (10.52)	5.71 (2.83 to 8.60)	<.001	0.66

^a^PHQ-9: Patient Health Questionnaire–9.

^b^MADRS-S: Montgomery-Åsberg Depression Rating Scale–self-rating version.

^c^EQ-VAS: EuroQol Visual Analogue Scale.

^d^PCS12: physical component score of the Short Form 12.

^e^MCS12: mental component score of the Short Form 12.

**Figure 3:**
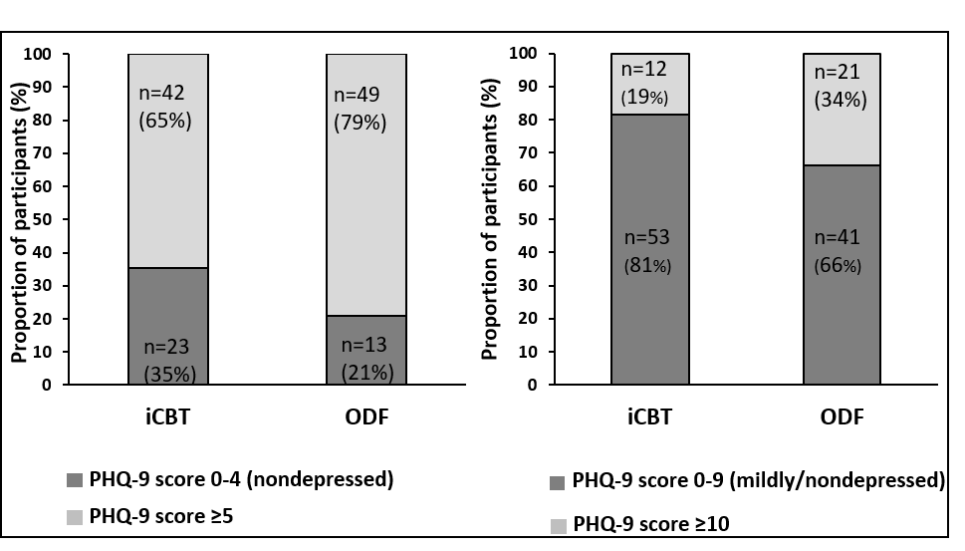
Proportion of patients according to dichotomized cutoff on Patient Health Questionnaire–9 (PHQ-9) in both groups at 9-week follow-up. Patients are grouped below the cutoff for nondepression and mild depression (PHQ-9 score 0-9) and below the cutoff for nondepression only (PHQ-9 score 0-4). iCBT (n=65): internet-based cognitive behavioral therapy; ODF (n=62): Web-based discussion forum.

In the per-protocol analysis (iCBT n=65 and ODF n=62), which aimed to compare categorical improvements in depression at 9-week follow-up, the proportion of patients who had a clinically significant improvement in depression (ie, a decrease of ≥5 points in PHQ-9) was larger in the iCBT group than in the ODF group (43%, n=28 vs 24%, n=15; *P*=.02). There was also a significantly larger proportion of nondepressed (PHQ-9 score <5; 35%, n=23 vs 21%, n=13; *P*=.02) or mildly/nondepressed (PHQ-9 score <10; 82%, n=53 vs 66%, n=41; *P*=.049; Figure 3) participants in the iCBT group compared with the ODF group. The NNT for a clinically significant change on PHQ-9 was 5. The NNT to become nondepressed was 7, and the NNT to become mildly depressed was 6. For MADRS-S, the NNT for iCBT was 3.

With regard to adherence, a total of 60% (n=43) of the iCBT group completed all 7 modules, whereas 82% (n=59) completed more than half (ie, 4 or more). In the ODF group, 27% (n=20) of the patients completed 9 or more activities (eg, reading or posting) in the forum threads. In the per-protocol analysis performed to compare the change in level of depression in relation to adherence (Multimedia Appendix 3), we first compared those who had completed at least one iCBT treatment module (n=69) with those with at least one activity in the ODF (n=49), in which a significant and moderate effect of iCBT was found (*P*<.001, Cohen *d*=0.69). In the next step, those in the iCBT group who had completed all 7 modules (n=43) were compared with those in the ODF who had at least 9 activities (n=20) and a significant and large effect of iCBT (*P*=.002, Cohen *d*=0.89) was found. The total mean time required for providing feedback to patients completing 7 modules was 113.8 (SD 46.3) min or an average of 13 min per week per patient.

With regard to safety, 1 patient in the iCBT group and 3 in the ODF group demonstrated an increase of more than 5 points on PHQ-9 when comparing individual baseline and 9-week follow-up measures. At baseline, 3 patients in each group reported a score of 2 or more on item 9 in PHQ-9 (thought about being better off dead). At 9 weeks, these numbers had decreased to 1 and 2 in the iCBT and ODF groups, respectively. On 2 occasions, 1 patient in the iCBT group scored above 4 on MADRS-S item 9 (zest for life) during the predefined weekly safety measures. The corresponding numbers for the ODF group were 3 occasions among 3 patients. These patients were contacted by telephone for an evaluation, but no patient was discontinued from the study owing to high risk of suicide or deterioration in depression.

## Discussion

### Principal Findings

To our knowledge, this is the first adequately powered RCT aimed at evaluating the effect of a nurse-delivered iCBT program for depression in patients diagnosed with CVD. We found that the program, which was adapted to fit the context of CVD, was more effective than the ODF in reducing depression and improving HRQoL.

We found a moderate effect for a nurse-delivered and adapted iCBT program on depression in patients with CVD (PHQ-9, Cohen *d*=0.62). However, measured by the MADRS-S, the effect on depression was strong (Cohen *d*=0.86). Furthermore, we also found an improved HRQoL, which is also of clinical relevance for the care of patients with chronic somatic disorders. The effects on depression are stronger than the reported small effect (standardized mean difference of 0.27) from the latest systematic review of psychological interventions for depression in patients with CVD [5]. However, our results correspond to the overall moderate effect size of 0.67 for depression reported in a recent meta-analysis [8] of iCBT studies, in which most of the patients did not have a chronic somatic disorder. The NNT in our study were 5 and 6 for a clinical improvement and being nondepressed, respectively. However, when we used MADRS-S, the NNT was 3 to become nondepressed. These NNT are comparable with the NNT of 4.6 reported in the same meta-analysis of studies using *care as usual* as a control group [8], although we used an active control group (ie, ODF), which we consider to be more active than many care-as-usual regimens. Furthermore, we included depressed patients, who thus had a risk of deterioration in depression. One strength of our study is that it was guided; the participants’ levels of depression were monitored and those with increasing scores and who were considered at risk of suicide were contacted for an assessment. This would not have been possible with an unguided program. Our results suggest that this nurse-led and adapted iCBT program was safe and provided effects on depression in patients with CVD that are comparable with the effects found in meta-analyses of iCBT studies [8].

The number of randomized controlled studies evaluating iCBT in CVD is surprisingly low. However, findings from a study [38] using a generic and unguided iCBT program on participants with self-reported high CVD risks and mild depression reported a small effect (*d*=0.15) on depression. A recent study from 2018, the U-CARE Heart study [39], reported that, surprisingly, a therapist-guided and adapted iCBT program for depression and anxiety in patients with a recent myocardial infarction (MI) was not superior to treatment as usual. Adherence in that study was low; 46% did not complete the first module and only 15% completed 2 or more. In our study, 60% completed all modules and 82% completed 4 or more. Moreover, the iCBT intervention seems to also be feasible for young and old patients with CVD. There are several possible explanations for why our iCBT program seems to be more effective and encourages greater adherence than the previously mentioned studies [38,39]. One explanation could be that the content of the iCBT program was adapted for CVD. In one study [40], depressed patients with heart failure did not describe the adapted iCBT program as a treatment for depression, but as a new way to actively learn self-care. In another study [41] conducted with patients with multiple sclerosis and comorbid depression, where iCBT was provided by a generic and automated program (ie, Deprexis), as many as 51% of the patients reported that the program required adjustments to fit the needs of patients with multiple sclerosis. On the other hand, the U-CARE [39] study used a therapist-guided and adapted iCBT program. However, in a qualitative study from U-CARE [42], the MI patients suggested that one improvement for that iCBT program could be to include the opportunity to ask medical questions to a health care professional. Thus, patients with chronic somatic disease and depression have a combination of medical and psychosocial needs that should be addressed in iCBT programs. Apart from tailoring the content, this also implies that those who deliver guidance can also address CVD-related issues when needed. Another possible explanation for our result is that feedback and support in our study were delivered by nurses with experience of CVD care, who had taken a short course in CBT. This corresponds to McCombie et al’s suggestion that iCBT programs for patients with physical illness should be adapted to each individual illness and provided by the medical team caring for the patient [13]. This may also facilitate the implementation of iCBT in clinical CVD care. An alternative adapted iCBT program could be designed to incorporate a collaboration between CVD nurses, psychologists, and cardiologists, who could all respond on-demand, depending on the needs of the patients.

### Strengths and Limitations

This study has several limitations. First, patients were included on the basis of self-reported depression and not on a diagnostic depression interview. This may have increased the risk of misclassification of depression. However, to increase the probability of the presence of at least mild depression, it was not enough to only have a score above 5 on the PHQ-9 during the screening but at least one of the two core symptoms of depression (depressed mood and/or loss of interest) was also necessary. Although it should not be considered as a full, formal diagnostic interview, MINI was used during the telephone interview to assess the presence of core symptoms, as well as the severity of the depression and any presence or history of suicidal ideation. Another limitation is that we did not include high alcohol consumption as a specific exclusion criterion. If there was high alcohol consumption that might hinder participation in the study, this was assessed by the study nurses during the telephone interview. One may also direct criticism toward the design of the ODF, as the activity in the ODF group may be considered low, with only about one-third completing 9 or more activities. The intention was that the discussion between members should be the motivating factor. However, as the ODF proceeded, the activity decreased. A potential explanation is that those in the ODF were not given feedback from the study team. One might argue that the ODF is no more than care as usual and that the effect of the iCBT is thus less valid. However, in the U-CARE study [39], the depression score in the iCBT group decreased by 3.3 points and by 2.3 points in the care-as-usual group. We therefore believe that the results reported here can be seen to provide important and valid information about the effects of the iCBT program on depression. All the study nurses had clinical experience of psychiatric care and were able to consult a psychologist or the other study members when needed. The recruitment of patients was challenging as we aimed to include patients with at least mild depression, which is not always the case in other psychological interventional studies of CVD [5]. We contacted 11,992 patients with CVD by mail. However, not all of these were expected to be eligible. We calculated that approximately 20% may be depressed. Thus, 2400 of those could be potential candidates to include in the study. A potential explanation for the challenge in recruiting participants may be that cardiac patients recognize electronic health or iCBT as something different from health care [40]. This may indicate that they did not value this type of intervention as a possibility to get help. In general terms, our recruitment process may be seen as valid as it corresponds to what is common in iCBT studies [38,39,43]. Despite our limitations in diagnosing depression, we therefore believe that our study contributes to the literature. ANCOVA is often used today when analyzing the outcome of RCT. In cases of missing data, ANCOVA is limited because there is currently no consensus about how to pool multiple imputed F-statistics, and we therefore used LOCF. We are fully aware of this limitation and therefore also provided a mixed-model analysis with multiple imputed data. Regardless of the type of statistical analysis, the result is maintained. One may argue that the need to have a computer and internet access may be a limiting factor in this study. iCBT will probably never fit all CVD patients, independent of whether they have a computer or internet access. Regarding access and using the internet, this is not a major problem in Sweden. Among those aged 56–65 years, only 2 % do not use the internet. Among those aged 66–75 years, 9 % do not use the internet. However, among those aged over 76 years, this rises to 42 %. The trend is, however, towards increasing use in all age groups within the Swedish population. Thus, iCBT can be seen as another tool in the management of depression and CVD. We argue that, if many CVD patients can be targeted with iCBT, there will also be a greater opportunity to provide face-to-face CBT to those CVD patients for whom iCBT will not fit. For those who had completed all seven modules, feedback was 13 min per week per patient. However, a limitation is that we do not have any information regarding the number of messages sent. All nurses who provided feedback were members of the study team, held specific competence in psychiatry and CVD and had a high interest in iCBT. This might be problematic if the iCBT program were to be implemented in clinical cardiac care since the ordinary healthcare personnel who may be expected to provide feedback will obviously not have all these specific competencies. However, the therapeutic part of iCBT is embedded within the text, whereas the feedback focuses more on general issues such as encouragement, confirming and reflection [9]. This suggests that feedback probably can be delivered by the vast majority of healthcare personnel within CVD care after a brief introduction to iCBT. To conclude, despite all the limitations in this study, we still believe that the results provide important and valid information about the effects of a nurse-delivered, adapted iCBT program designed to reduce depression in patients with CVD.

### Conclusions

This study has shown that a nurse-delivered and adapted iCBT program decreased depression and improved HRQoL in CVD patients with depression. A clear majority of the patients also adhered to most parts of the iCBT program. Our findings suggest that the implementation of iCBT in CVD care means that more patients, who at present might not receive adequate treatment, can now gain access to psychological treatment for depression in their own homes and at a time of their own choosing.

## Appendix

Multimedia Appendix 1 Home and log in site.

Multimedia Appendix 2 Description of the iCBT program and examples of homework assignments.

Multimedia Appendix 3 Supplementary results.

Multimedia Appendix 4 CONSORT‐EHEALTH checklist (V 1.6.1).

## Abbreviations

ANCOVA: analysis of covariance
CBT: cognitive behavioral therapy
CVD: cardiovascular disease
EQ-VAS: EuroQol Visual Analogue Scale
HRQoL: health-related quality of life
iCBT: internet-based cognitive behavioral therapy
ICD: International Classification of Diseases
LOCF: last observation carried forward
MADRS-S: Montgomery-Åsberg Depression Rating Scale–self-rating Scale
MCS: mental component score
MI: myocardial infarction
MINI: Mini International Neuropsychiatric Interview
NNT: numbers needed to treat
NYHA: New York Heart Association
ODF: online discussion forum
PCS: physical component score
PHQ-9: Patient Health Questionnaire–9
RCT: randomized controlled trial
SF-12: Short Form 12
