# Supplementary figures and images for: An Internet-Based Cognitive Behavioral Therapy Program Adapted to Patients With Cardiovascular Disease and Depression: Randomized Controlled Trial
Source: JMIR Ment Health. 2019 Oct 3;6(10):e14648. doi: 10.2196/14648 (PMC7020777; doi:10.2196/14648)

## Slide 1
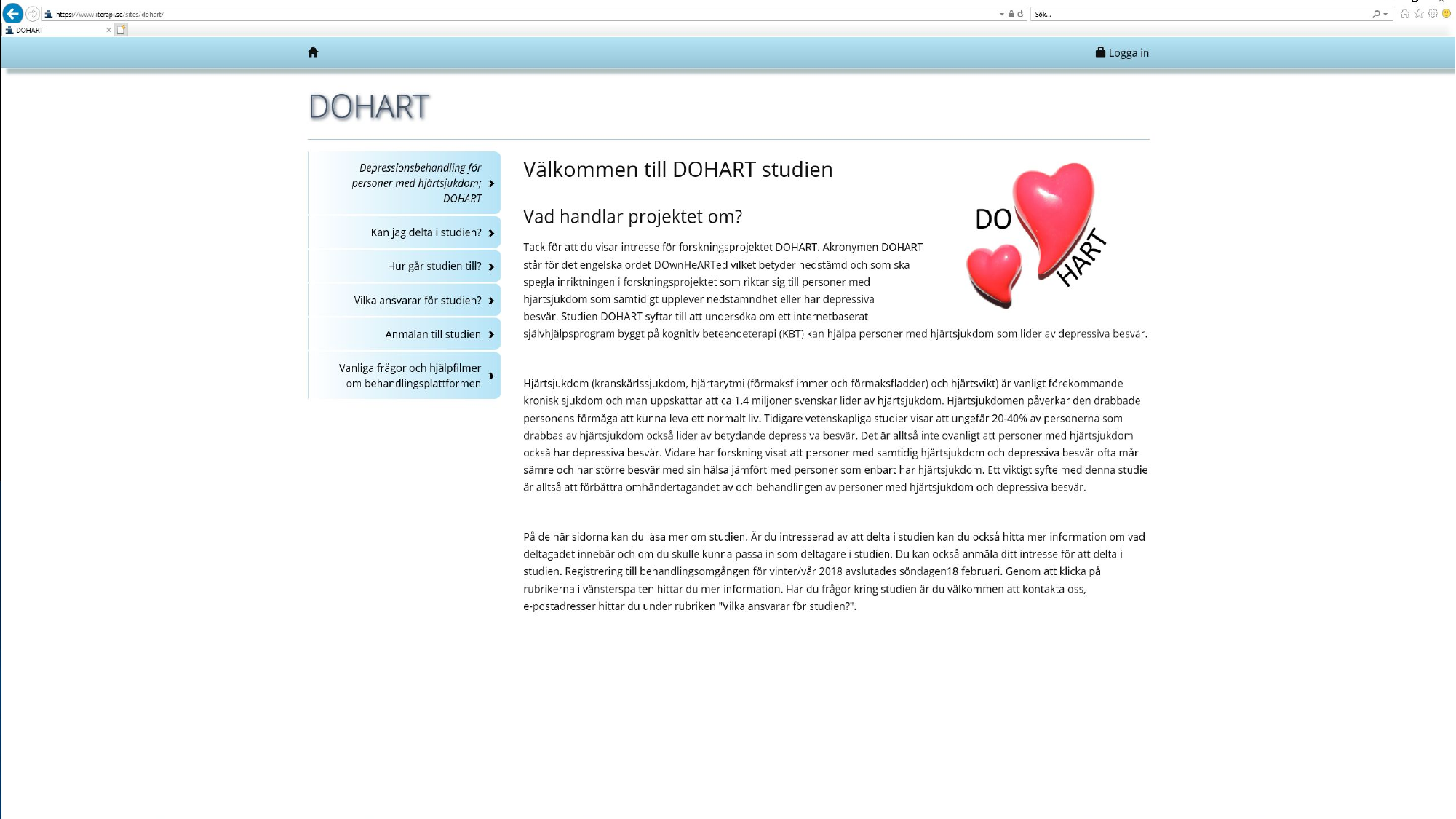

Supplement: Multimedia Appendix 1 [file mental_v6i10e14648_app1.pptx]

## Slide 1
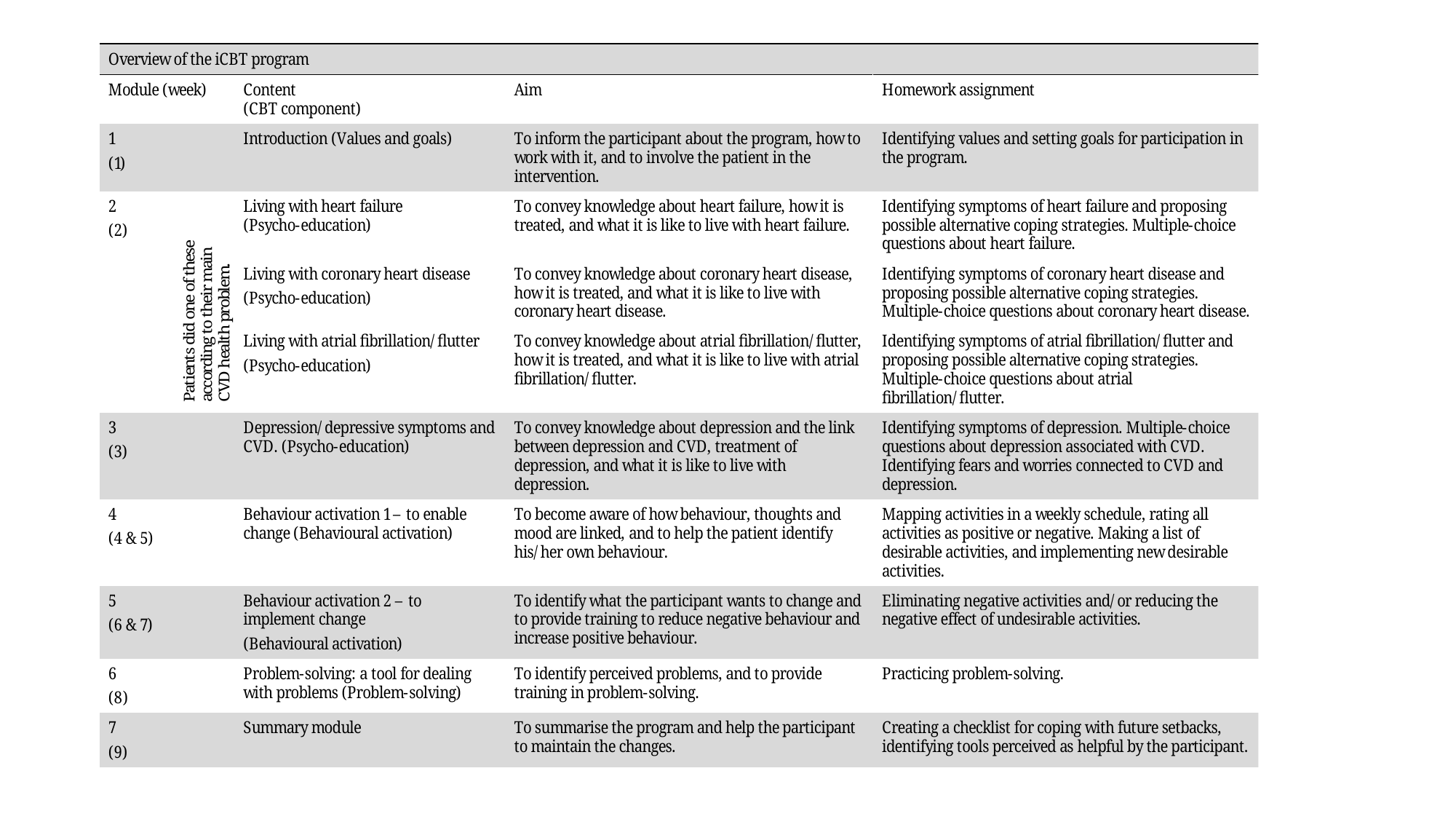

## Slide 2
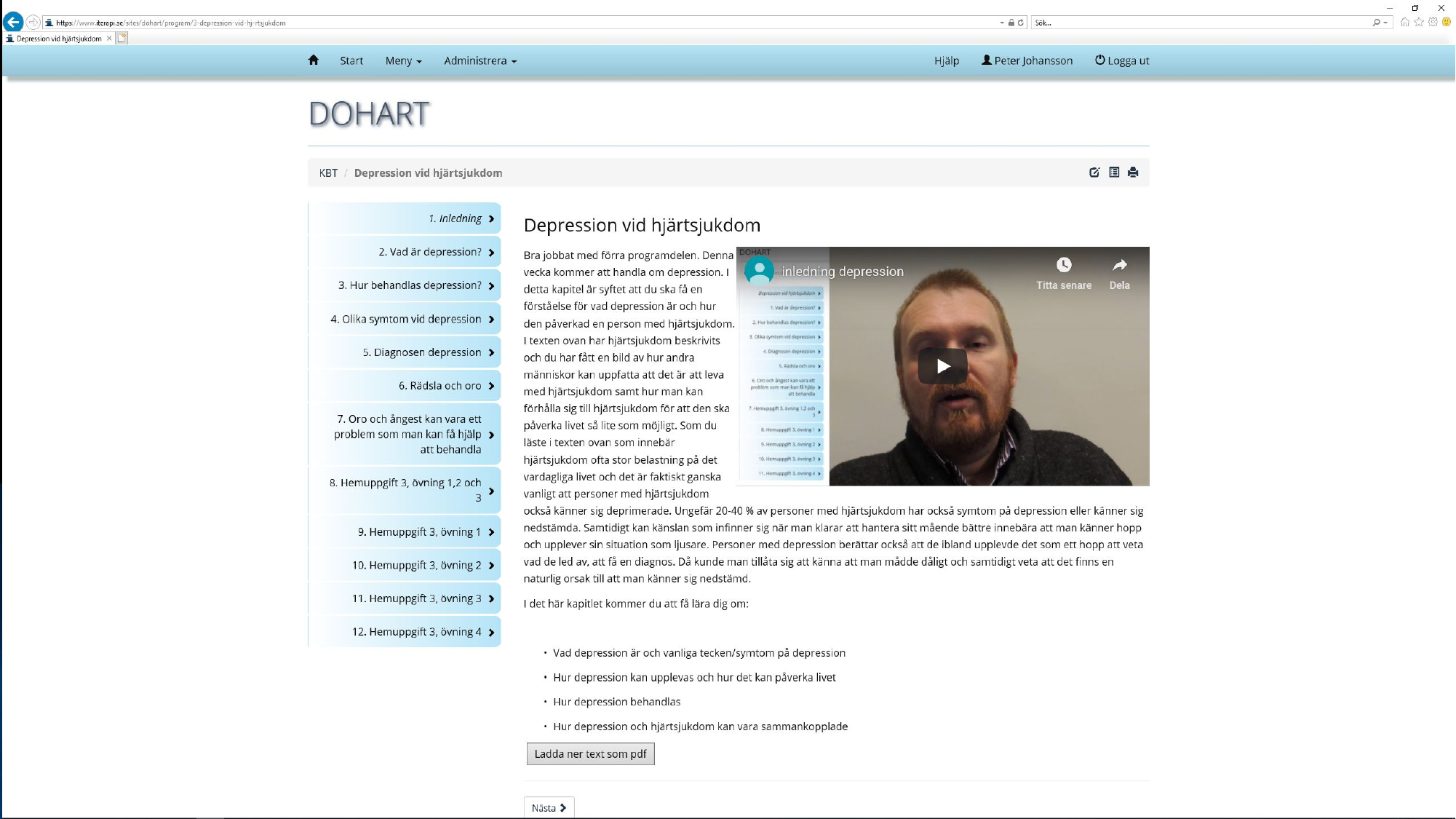

## Slide 3
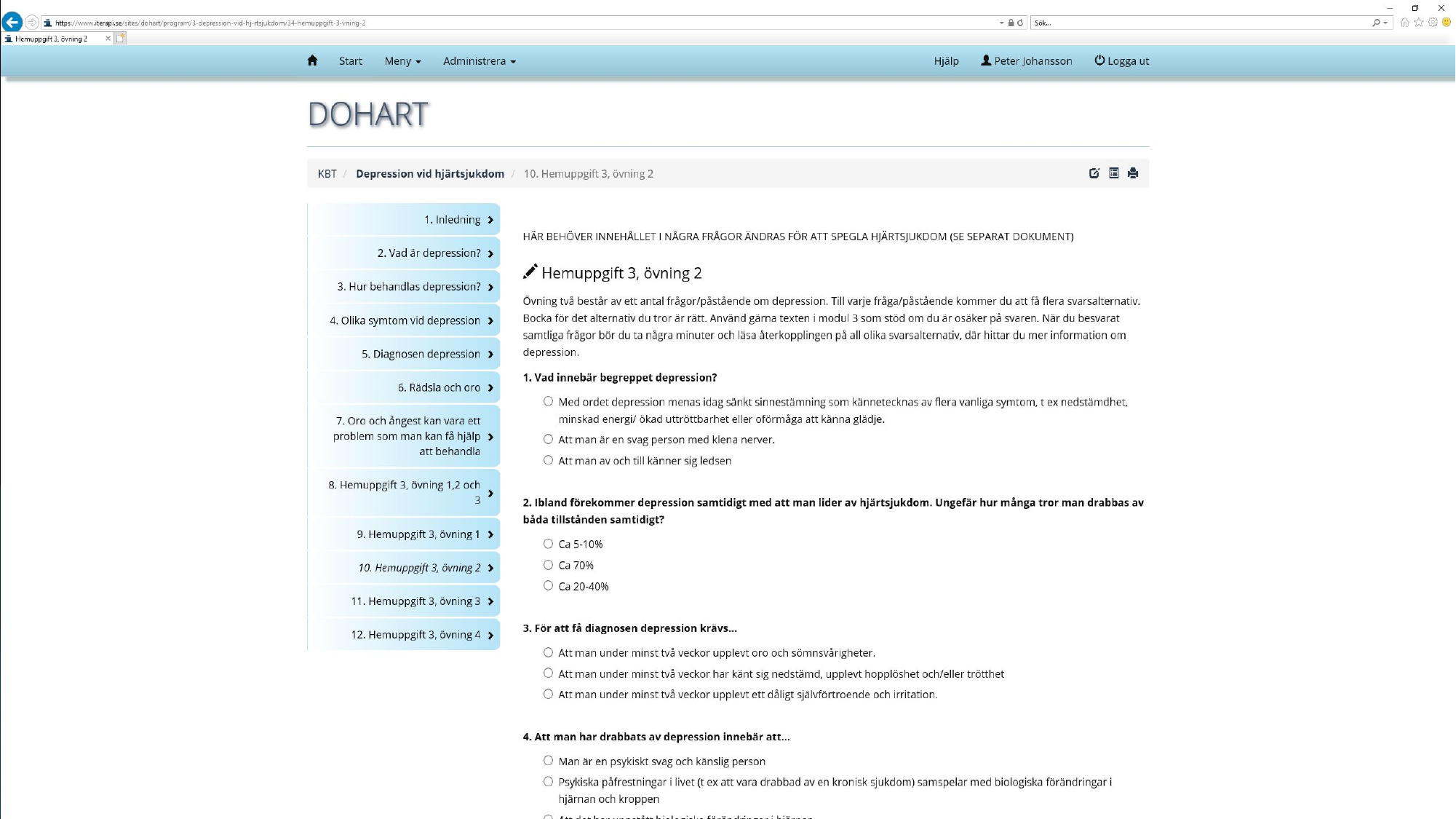

## Slide 4
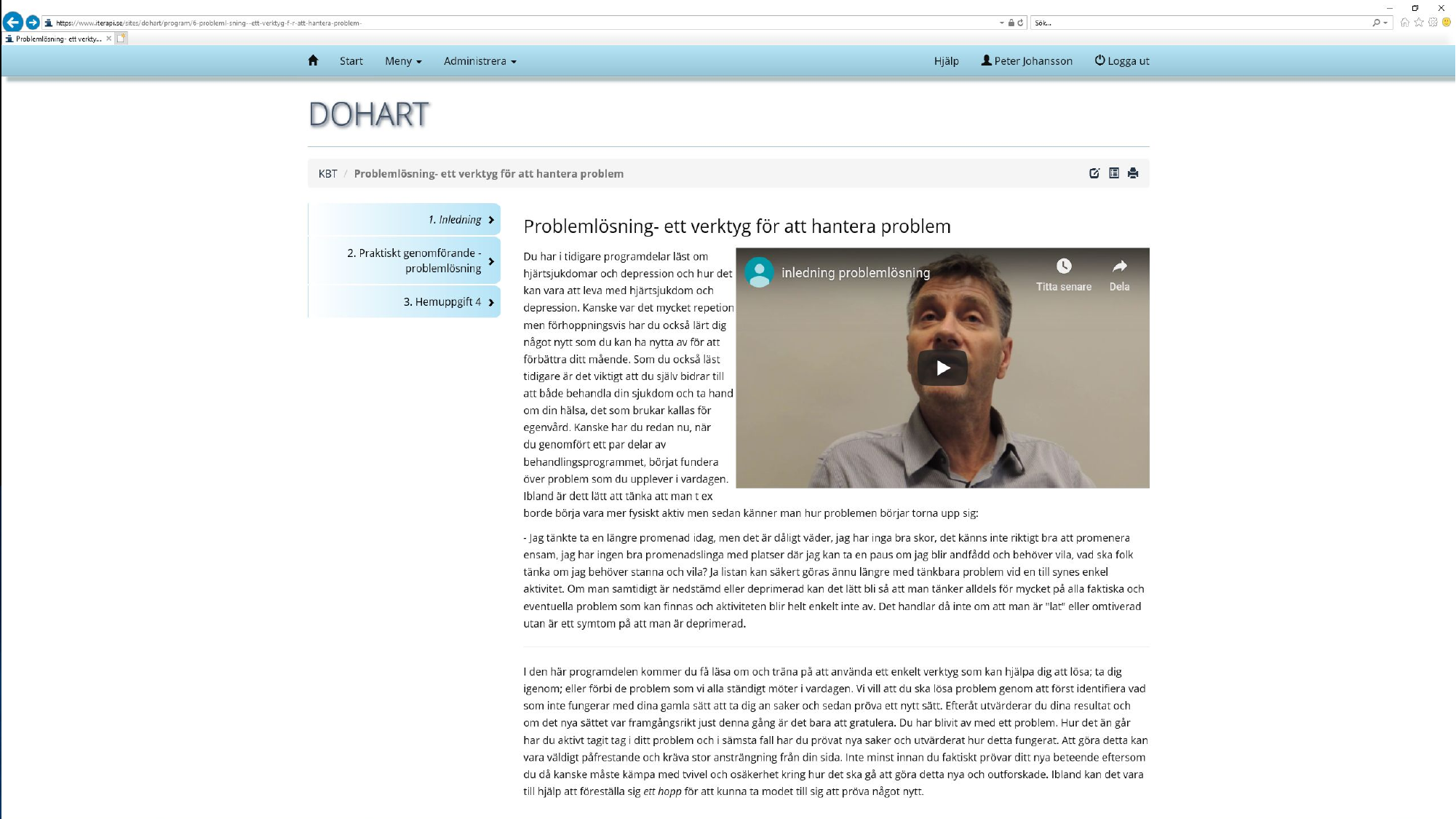

## Slide 5
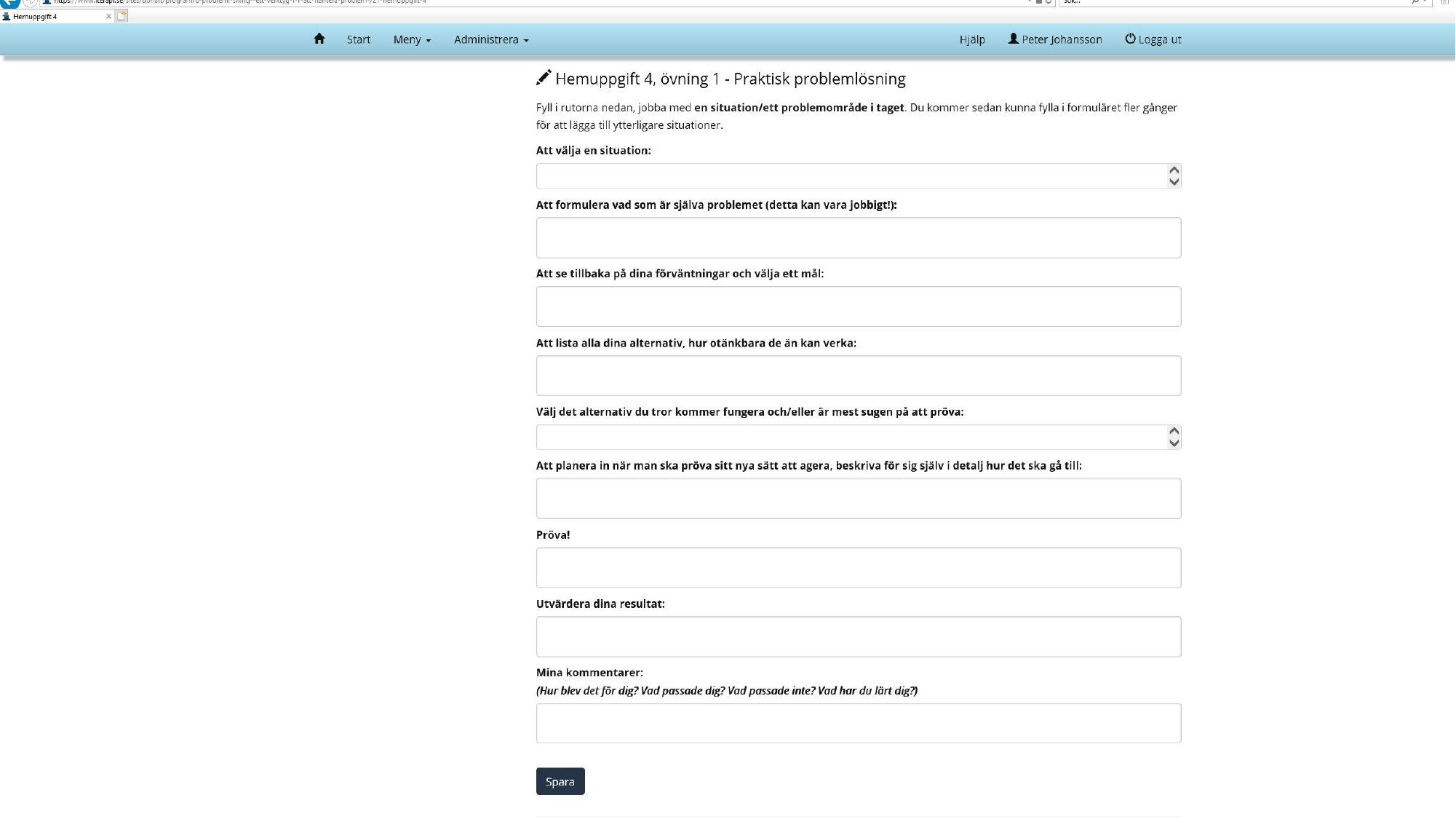

Supplement: Multimedia Appendix 2 [file mental_v6i10e14648_app2.pptx]
