# Supplementary material for: An Internet-Based Cognitive Behavioral Therapy Program Adapted to Patients With Cardiovascular Disease and Depression: Randomized Controlled Trial
Source: JMIR Ment Health. 2019 Oct 3;6(10):e14648. doi: 10.2196/14648 (PMC7020777; doi:10.2196/14648)
Supplement: Multimedia Appendix 3 [file mental_v6i9e14648_app3.pdf]

Multimedia Appendix 3

Supplementary results to

[The effect of an internet-based cognitive behavioural therapy programme adapted to patients with cardiovascular disease and depression – a randomised controlled study]

Johansson, P., Westas, M., Andersson, G., Alehagen, U.,
Broström, A., Jaarsma, T., Mourad, G., Lundgren, J.

Results based on mixed model analysis using multiple imputed data.

The final mixed model analysis on multiple imputed data showed a significant effect of iCBT compared to ODF on PHQ-9 (*P*=.002), MADRS-S (*P* <.001.), EQ-VAS (*P* <.001.) and MCS (*P* <.001.) There was no significant difference between iCBT and ODF with regard to PCS (*P*=.26). Effect sizes calculated on multiple imputed data indicate moderate effects of iCBT on depression and HRQoL, (Table A1).

Table A1: Treatment effects

All data is imputed using multiple imputation. Data are mean (SD) unless otherwise stated. SD is pooled in accordance with the following equation: $S_{pooled} = \sqrt{\frac{(n_1-1)s_1^2+(n_2-1)s_2^2+\cdots+(n_k-1)s_k^2}{n_1+n_2+\cdots+n_k-k}}$

	**iCBT (n=72)**		**ODF (n=72)**		**Estimate for iCBT and time interaction, 95% CI**		***P***	**Effect size (d)**
	Baseline	9 weeks	Baseline	9 weeks				
PHQ-9	10.71 (4.47)	6.39 (4.71)	10.22 (5.19)	8.30 (4.65)	-2.40	-3.93 to -0.87	.002	0.51
MADRS-S	18.01 (7.25)	10.57 (7.51)	17.67 (6.31)	15.60 (8.26)	-5.38	-7.84 to -2.92	<.001.	0.71
EQ-VAS·	53.56 (20.09)	65.34 (21.84)	57.36 (18.47)	56.27 (22.72)	12.86	6.29 to 19.44	<.001.	0.64
PCS12	39.80 (10.09)	42.05 (10.63)	37.79 (11.22)	38.32 (11.71)	1.72	-1.28 to 4.72	.26	0.19
MCS12	36.08 (9.36)	43.62 (11.37)	36.41 (10.00)	38.18 (11.00)	5.77	2.39 to 9.16	<.001.	0.56

PHQ-9= Patient Health Questionnaire 9. MADRS-S= Montgomery Åsberg Depression Rating Scale (self-rating version). EQ-VAS = EuroQol Visual Analogue Scale. PCS12= Physical Component Score of the Short Form 12. MCS12= Mental Componet Score of the Short Form 12.

Per protocol analyses

The per-protocol analysis was divided into two parts. In the first analysis all participants in the iCBT group that had completed at least one module in iCBT (n=64) was compared to those who had performed at least one activity in the ODF (n=46) (Table A2). This analysis showed a significant effect of iCBT on depression (PHQ-9 mean between group difference -2.72 (*P*<.001.) with a moderate effect size (d=0.69). Depression as measured by MADRS-S showed a significant (*P*<.001.) and large effect size (0.87). HRQoL as measured by EQ-VAS

and MCS showed a significant difference favouring iCBT (*P*<.001.) and, *P*<.001.) For the PCS no significant difference was found (p=.39).

Table A2: Treatment effect per-protocol

Observed data on participants with at least one iCBT module or ODF activity. Data are mean (SD) unless otherwise stated.

	**iCBT (n=64)**		**ODF (n=46)**		**Mean between-group treatment difference (95% CI)**		***P***	**Effect size (d)**
	Baseline	9 weeks	Baseline	9 weeks				
PHQ-9	10.74 (4.56)	6.30 (4.79)	10.14 (5.11)	8.63 (4.53)	-2.72	-4.24 to -1.20	<.001.	0.69
MADRS-S	18.19 (6.96)	10.30 (7.42)	17.86 (5.76)	16.21 (8.34)	-5.95	-8.56 to -3.34	<.001.	0.87
EQ-VAS	53.12 (20.22)	66.14 (21.69)	58.73 (17.87)	58.46 (21.92)	11.56	4.88 to 18.25	<.001.	0.66
PCS12	39.79 (10.16)	42.38 (10.74)	39.98 (10.32)	41.26 (11.06)	1.31	-1.76 to 4.38	.39	0.16
MCS12	35.80 (9.33)	43.94 (11.41)	36.45 (10.49)	36.81 (10.75)	7.17	3.70 to 10.63	<.001.	0.79

PHQ-9= Patient Health Questionnaire 9. MADRS-S= Montgomery Åsberg Depression Rating Scale (self-rating version). EQ-VAS = EuroQol Visual Analogue Scale. PCS= Physical Component Score of the Short Form 12. MCS= Mental Component Score of the Short Form 12.

In the second per-protocol analysis those participants in the iCBT group that had completed the full programme (i.e. seven modules, n=43) were compared to those in the ODF (n=20) who had performed at least nine activities (n=20) (Table A3). This analysis revealed a significant effect of iCBT on depression (PHQ-9 mean between group difference -3·56 (*P*=.002) with a large effect size (d=0.89). Depression as measured by MADRS-S showed a significant (*P*=.002) and large effect size (.90). HRQoL as measured by EQ-VAS and MCS showed a significant difference favouring iCBT (*P*<.001. and) *P*<.001. As for the PCS no significant difference was found (*P*=.26).

Table A3. Treatment effect per-protocol

Observed data on participants with seven iCBT modules or at least nine ODF activities. Data are mean (SD) unless otherwise stated.

	**iCBT (n=43)**		**ODF (n=20)**		**Mean between-group treatment difference (95% CI)**		***P***	**Effect size (d)**
	Baseline	9 weeks	Baseline	9 weeks				
PHQ-9	10.86 (4.87)	6.09 (5.11)	11.15 (6.07)	9.80 (4.00)	-3.56	-5.74 to -1.37	.002	0.89
MADRS-S	18.12 (7.16)	9.88 (7.95)	20.00 (6.77)	17.35 (8.67)	-6.29	-10.08 to -2.50	.002	0.90
EQ-VAS	50.35 (20.19)	66.12 (22.80)	55.90 (16.89)	52.15 (22.27)	18.19	8.67 to 27.71	<.001.	1.03
PCS12	38.21 (8.79)	41.96 (11.04)	39.93 (11.07)	40.73 (12.44)	2.62	-2.01 to 7.25	.26	0.31
MCS12	35.90 (9.83)	44.93 (12.28)	34.34 (10.06)	34.10 (8.54)	9.80	4.78 to 14.83	<.001.	1.06

PHQ-9= Patient Health Questionnaire 9. MADRS-S= Montgomery Åsberg Depression Rating Scale (self-rating version). EQ-VAS = EuroQol Visual Analogue Scale. PCS12= Physical Component Score of the Short Form 12. MCS12= Mental Component Score of the Short Form 12.
