# Supplementary material for: An Internet-Based Cognitive Behavioral Therapy Program Adapted to Patients With Cardiovascular Disease and Depression: Randomized Controlled Trial
Source: JMIR Ment Health. 2019 Oct 3;6(10):e14648. doi: 10.2196/14648 (PMC7020777; doi:10.2196/14648)
Supplement: Multimedia Appendix 4 [file mental_v6i10e14648_app4.pdf]

CONSORT-EHEALTH (V 1.6.1) - Submission/Publication Form

The CONSORT-EHEALTH checklist is intended for authors of randomized trials evaluating web-based and Internet-based applications/interventions, including mobile interventions, electronic games (incl multiplayer games), social media, certain telehealth applications, and other interactive and/or networked electronic applications. Some of the items (e.g. all subitems under item 5 - description of the intervention) may also be applicable for other study designs.

The goal of the CONSORT EHEALTH checklist and guideline is to be
a) a guide for reporting for authors of RCTs,
b) to form a basis for appraisal of an ehealth trial (in terms of validity)

CONSORT-EHEALTH items/subitems are MANDATORY reporting items for studies published in the Journal of Medical Internet Research and other journals / scientific societies endorsing the checklist.

Items numbered 1., 2., 3., 4a., 4b etc are original CONSORT or CONSORT-NPT (non-pharmacologic treatment) items.
Items with Roman numerals (i., ii, iii, iv etc.) are CONSORT-EHEALTH extensions/clarifications.

As the CONSORT-EHEALTH checklist is still considered in a formative stage, we would ask that you also RATE ON A SCALE OF 1-5 how important/useful you feel each item is FOR THE PURPOSE OF THE CHECKLIST and reporting guideline (optional).

Mandatory reporting items are marked with a red *.
In the textboxes, either copy & paste the relevant sections from your manuscript into this form - please include any quotes from your manuscript in QUOTATION MARKS,
or answer directly by providing additional information not in the manuscript, or elaborating on why the item was not relevant for this study.

YOUR ANSWERS WILL BE PUBLISHED AS A SUPPLEMENTARY FILE TO YOUR PUBLICATION IN JMIR AND ARE CONSIDERED PART OF YOUR PUBLICATION (IF ACCEPTED).
Please fill in these questions diligently. Information will not be copyedited, so please use proper spelling and grammar, use correct capitalization, and avoid abbreviations.

DO NOT FORGET TO SAVE AS PDF _AND_ CLICK THE SUBMIT BUTTON SO YOUR ANSWERS ARE IN OUR DATABASE !!!

Citation Suggestion (if you append the pdf as Appendix we suggest to cite this paper in the caption):
Eysenbach G, CONSORT-EHEALTH Group
CONSORT-EHEALTH: Improving and Standardizing Evaluation Reports of Web-based and Mobile Health Interventions
J Med Internet Res 2011;13(4):e126
URL: http://www.jmir.org/2011/4/e126/
doi: 10.2196/jmir.1923
PMID: 22209829

*Obligatorisk

Your name *
First Last

Peter Johansson

Primary Affiliation (short), City, Country *
University of Toronto, Toronto, Canada

Linköping University

Your e-mail address *
abc@gmail.com

peter.b.johansson@liu.se

Title of your manuscript *

Provide the (draft) title of your manuscript.

Internet-based cognitive behavioural therapy programme tailored to patients with cardiovascular disease and depression: a randomised controlled trial

Name of your App/Software/Intervention *

If there is a short and a long/alternate name, write the short name first and add the long name in brackets.

DOHART (Downhearted)

Evaluated Version (if any)

e.g. "V1", "Release 2017-03-01", "Version 2.0.27913"

Ditt svar

Language(s) *

What language is the intervention/app in? If multiple languages are available, separate by comma (e.g. "English, French")

Swedish

URL of your Intervention Website or App

e.g. a direct link to the mobile app on app in appstore (itunes, Google Play), or URL of the website. If the intervention is a DVD or hardware, you can also link to an Amazon page.

https://www.iterapi.se/sites/dohart/

URL of an image/screenshot (optional)

Ditt svar

Accessibility *

Can an enduser access the intervention presently?

- ○ access is free and open
- ◉ access only for special usergroups, not open
- ○ access is open to everyone, but requires payment/subscription/in-app purchases
- ○ app/intervention no longer accessible
- ○ Övrigt:

Primary Medical Indication/Disease/Condition *

e.g. "Stress", "Diabetes", or define the target group in brackets after the condition, e.g. "Autism (Parents of children with)", "Alzheimers (Informal Caregivers of)"

Cardiovascular disease and depression

Primary Outcomes measured in trial *

comma-separated list of primary outcomes reported in the trial

Depression

Secondary/other outcomes
Are there any other outcomes the intervention is expected to affect?

Health-related quality of Life, Adherence

Recommended "Dose" *
What do the instructions for users say on how often the app should be used?

- ○ Approximately Daily
- ◉ Approximately Weekly
- ○ Approximately Monthly
- ○ Approximately Yearly
- ○ "as needed"
- ○ Övrigt:

Approx. Percentage of Users (starters) still using the app as recommended after 3 months *

- ○ unknown / not evaluated
- ○ 0-10%
- ○ 11-20%
- ○ 21-30%
- ○ 31-40%
- ○ 41-50%
- ○ 51-60%
- ○ 61-70%
- ○ 71%-80%
- ○ 81-90%
- ○ 91-100%
- ◉ Övrigt: Not applicable since iCBT program was nine weeks

Overall, was the app/intervention effective? *

- ◉ yes: all primary outcomes were significantly better in intervention group vs control
- ○ partly: SOME primary outcomes were significantly better in intervention group vs control
- ○ no statistically significant difference between control and intervention
- ○ potentially harmful: control was significantly better than intervention in one or more outcomes
- ○ inconclusive: more research is needed
- ○ Övrigt:

Article Preparation Status/Stage *
At which stage in your article preparation are you currently (at the time you fill in this form)

- ○ not submitted yet - in early draft status
- ◉ not submitted yet - in late draft status, just before submission
- ○ submitted to a journal but not reviewed yet
- ○ submitted to a journal and after receiving initial reviewer comments
- ○ submitted to a journal and accepted, but not published yet
- ○ published
- ○ Övrigt:

Journal *
If you already know where you will submit this paper (or if it is already submitted), please provide the journal name (if it is not JMIR, provide the journal name under "other")

- ○ not submitted yet / unclear where I will submit this
- ◉ Journal of Medical Internet Research (JMIR)
- ○ JMIR mHealth and UHealth
- ○ JMIR Serious Games
- ○ JMIR Mental Health
- ○ JMIR Public Health
- ○ JMIR Formative Research
- ○ Other JMIR sister journal
- ○ Övrigt:

Is this a full powered effectiveness trial or a pilot/feasibility trial? *

- ○ Pilot/feasibility
- ◉ Fully powered

Manuscript tracking number *
If this is a JMIR submission, please provide the manuscript tracking number under "other" (The ms tracking number can be found in the submission acknowledgement email, or when you login as author in JMIR. If the paper is already published in JMIR, then the ms tracking number is the four-digit number at the end of the DOI, to be found at the bottom of each published article in JMIR)

- ◉ no ms number (yet) / not (yet) submitted to / published in JMIR
- ○ Övrigt:

TITLE AND ABSTRACT

1a) TITLE: Identification as a randomized trial in the title

1a) Does your paper address CONSORT item 1a? *

I.e does the title contain the phrase "Randomized Controlled Trial"? (if not, explain the reason under "other")

- (selected) yes
- Övrigt:

1a-i) Identify the mode of delivery in the title

Identify the mode of delivery. Preferably use "web-based" and/or "mobile" and/or "electronic game" in the title. Avoid ambiguous terms like "online", "virtual", "interactive". Use "Internet-based" only if Intervention includes non-web-based Internet components (e.g. email), use "computer-based" or "electronic" only if offline products are used. Use "virtual" only in the context of "virtual reality" (3-D worlds). Use "online" only in the context of "online support groups". Complement or substitute product names with broader terms for the class of products (such as "mobile" or "smart phone" instead of "iphone"), especially if the application runs on different platforms.

subitem not at all important ○ ○ ○ ○ ● essential

Does your paper address subitem 1a-i? *

Copy and paste relevant sections from manuscript title (include quotes in quotation marks "like this" to indicate direct quotes from your manuscript), or elaborate on this item by providing additional information not in the ms, or briefly explain why the item is not applicable/relevant for your study

Internet-based cognitive behavioural therapy programme tailored to patients with cardiovascular disease and depression: a randomised controlled trial

1a-ii) Non-web-based components or important co-interventions in title

Mention non-web-based components or important co-interventions in title, if any (e.g., "with telephone support").

subitem not at all important ○ ○ ● ○ ○ essential

Does your paper address subitem 1a-ii?

Copy and paste relevant sections from manuscript title (include quotes in quotation marks "like this" to indicate direct quotes from your manuscript), or elaborate on this item by providing additional information not in the ms, or briefly explain why the item is not applicable/relevant for your study

Our intervention does not include a non web-based component

1a-iii) Primary condition or target group in the title

Mention primary condition or target group in the title, if any (e.g., "for children with Type I Diabetes") Example: A Web-based and Mobile Intervention with Telephone Support for Children with Type I Diabetes: Randomized Controlled Trial

subitem not at all important ○ ○ ○ ○ ● essential

Does your paper address subitem 1a-iii? *

Copy and paste relevant sections from manuscript title (include quotes in quotation marks "like this" to indicate direct quotes from your manuscript), or elaborate on this item by providing additional information not in the ms, or briefly explain why the item is not applicable/relevant for your study

Internet-based cognitive behavioural therapy programme tailored to patients with cardiovascular disease and depression: a randomised controlled trial

1b) ABSTRACT: Structured summary of trial design, methods, results, and conclusions

NPT extension: Description of experimental treatment, comparator, care providers, centers, and blinding status.

1b-i) Key features/functionalities/components of the intervention and comparator in the METI

Mention key features/functionalities/components of the intervention and comparator in the abstract. If possible, also mention theories and principles used for designing the site. Keep in mind the needs of systematic reviewers and indexers by including important synonyms. (Note: Only report in the abstract what the main paper is reporting. If this information is missing from the main body of text, consider adding it)

subitem not at all important ○ ○ ○ ○ ◉ essential

Does your paper address subitem 1b-i? *

Copy and paste relevant sections from the manuscript abstract (include quotes in quotation marks "like this" to indicate direct quotes from your manuscript), or elaborate on this item by providing additional information not in the ms, or briefly explain why the item is not applicable/relevant for your study

A randomised controlled trial. 144 CVD patients with at least mild depression (Patient Health Questionnaire-9 (PHQ-9) score ≥ 5) were randomised 1.1 to nine-week iCBT (n=72) or an active control participating in a Web-based discussion forum (ODF, n=72).

1b-ii) Level of human involvement in the METHODS section of the ABSTRACT

Clarify the level of human involvement in the abstract, e.g., use phrases like “fully automated” vs. “therapist/nurse/care provider/physician-assisted” (mention number and expertise of providers involved, if any). (Note: Only report in the abstract what the main paper is reporting. If this information is missing from the main body of text, consider adding it)

subitem not at all important ○ ○ ◉ ○ ○ essential

Does your paper address subitem 1b-ii?

Copy and paste relevant sections from the manuscript abstract (include quotes in quotation marks "like this" to indicate direct quotes from your manuscript), or elaborate on this item by providing additional information not in the ms, or briefly explain why the item is not applicable/relevant for your study

To evaluate the effect of a nurse-delivered and tailored internet-based cognitive behavioural therapy (iCBT) programme aimed at reducing depression in patients with CVD

1b-iii) Open vs. closed, web-based (self-assessment) vs. face-to-face assessments in the MET

Mention how participants were recruited (online vs. offline), e.g., from an open access website or from a clinic or a closed online user group (closed usergroup trial), and clarify if this was a purely web-based trial, or there were face-to-face components (as part of the intervention or for assessment). Clearly say if outcomes were self-assessed through questionnaires (as common in web-based trials). Note: In traditional offline trials, an open trial (open-label trial) is a type of clinical trial in which both the researchers and participants know which treatment is being administered. To avoid confusion, use “blinded” or “unblinded” to indicated the level of blinding instead of “open”, as “open” in web-based trials usually refers to “open access” (i.e. participants can self-enrol). (Note: Only report in the abstract what the main paper is reporting. If this information is missing from the main body of text, consider adding it)

subitem not at all important ○ ◉ ○ ○ ○ essential

Does your paper address subitem 1b-iii?

Copy and paste relevant sections from the manuscript abstract (include quotes in quotation marks "like this" to indicate direct quotes from your manuscript), or elaborate on this item by providing additional information not in the ms, or briefly explain why the item is not applicable/relevant for your study

We have included this information in the methods section of the manuscript. On page 5 we describe that ". Patients interested in study participation were instructed to register on the study website (www.dohart.se) (see screenshot in Multimedia appendix 1), which is a secure website requiring two-factor authentication to access the study platform [14]". On page 6 we describe "Patients who had registered on the study website were asked to complete a web-based screening form that collected data about depression as assessed by PHQ, demographics, smoking and alcohol habits, CVD diagnosis, time since diagnosis of CVD, NYHA classification, comorbidities and medications for CVD, depression, sleep problems and anxiety."

1b-iv) RESULTS section in abstract must contain use data

Report number of participants enrolled/assessed in each group, the use/uptake of the intervention (e.g., attrition/adherence metrics, use over time, number of logins etc.), in addition to primary/secondary outcomes. (Note: Only report in the abstract what the main paper is reporting. If this information is missing from the main body of text, consider adding it)

subitem not at all important ○ ○ ○ ○ ◉ essential

Does your paper address subitem 1b-iv?

Copy and paste relevant sections from the manuscript abstract (include quotes in quotation marks "like this" to indicate direct quotes from your manuscript), or elaborate on this item by providing additional information not in the ms, or briefly explain why the item is not applicable/relevant for your study

"144 CVD patients with at least mild depression (Patient Health Questionnaire-9 (PHQ-9) score ≥ 5) were randomised 1.1 to nine-week iCBT (n=72) or an active control participating in a Web-based discussion forum (ODF, n=72)."

1b-v) CONCLUSIONS/DISCUSSION in abstract for negative trials

Conclusions/Discussions in abstract for negative trials: Discuss the primary outcome - if the trial is negative (primary outcome not changed), and the intervention was not used, discuss whether negative results are attributable to lack of uptake and discuss reasons. (Note: Only report in the abstract what the main paper is reporting. If this information is missing from the main body of text, consider adding it)

subitem not at all important ○ ○ ○ ◉ ○ essential

Does your paper address subitem 1b-v?

Copy and paste relevant sections from the manuscript abstract (include quotes in quotation marks "like this" to indicate direct quotes from your manuscript), or elaborate on this item by providing additional information not in the ms, or briefly explain why the item is not applicable/relevant for your study

Our trial had statistically significant and positive results.

INTRODUCTION

2a) In INTRODUCTION: Scientific background and explanation of rationale

2a-i) Problem and the type of system/solution

Describe the problem and the type of system/solution that is object of the study: intended as stand-alone intervention vs. incorporated in broader health care program? Intended for a particular patient population? Goals of the intervention, e.g., being more cost-effective to other interventions, replace or complement other solutions? (Note: Details about the intervention are provided in “Methods” under 5)

subitem not at all important ○ ○ ○ ◉ ○ essential

Does your paper address subitem 2a-i? *

Copy and paste relevant sections from the manuscript (include quotes in quotation marks "like this" to indicate direct quotes from your manuscript), or elaborate on this item by providing additional information not in the ms, or briefly explain why the item is not applicable/relevant for your study

"A possible solution could be iCBT, which can be provided at a low cost and has been proved effective in patients with depression [7, 8]. iCBT can be delivered as guided (i.e. support and/or encouragement and/or feedback on homework assignments [9]) or unguided, but guided iCBT tends to be more effective [10]. One important aspect that may facilitate the implementation of guided iCBT in clinical care is that it can be delivered by healthcare professionals with little or no training in iCBT, without reducing the effect of the treatment [11]. Another advantage of iCBT is that it enables CVD patients’ access to CBT in their own homes and at a time that is suitable to them. However, generic iCBT programmes may not be optimal for targeting depression in patients with chronic diseases, since these programmes are not tailored to the context of the disease [12, 13]. In the present study, we therefore aim to evaluate the effect of a nurse-delivered tailored iCBT programme to reduce depression in patients with CVD."

2a-ii) Scientific background, rationale: What is known about the (type of) system

Scientific background, rationale: What is known about the (type of) system that is the object of the study (be sure to discuss the use of similar systems for other conditions/diagnoses, if appropiate), motivation for the study, i.e. what are the reasons for and what is the context for this specific study, from which stakeholder viewpoint is the study performed, potential impact of findings [2]. Briefly justify the choice of the comparator.

subitem not at all important ○ ○ ◉ ○ ○ essential

Does your paper address subitem 2a-ii? *

Copy and paste relevant sections from the manuscript (include quotes in quotation marks "like this" to indicate direct quotes from your manuscript), or elaborate on this item by providing additional information not in the ms, or briefly explain why the item is not applicable/relevant for your study

"iCBT can be delivered as guided (i.e. support and/or encouragement and/or feedback on homework assignments [9]) or unguided, but guided iCBT tends to be more effective [10]. One important aspect that may facilitate the implementation of guided iCBT in clinical care is that it can be delivered by healthcare professionals with little or no training in iCBT, without reducing the effect of the treatment [11]. Another advantage of iCBT is that it enables CVD patients’ access to CBT in their own homes and at a time that is suitable to them."

2b) In INTRODUCTION: Specific objectives or hypotheses

Does your paper address CONSORT subitem 2b? *

Copy and paste relevant sections from the manuscript (include quotes in quotation marks "like this" to indicate direct quotes from your manuscript), or elaborate on this item by providing additional information not in the ms, or briefly explain why the item is not applicable/relevant for your study

"In the present study, we therefore aim to evaluate the effect of a nurse-delivered tailored iCBT programme to reduce depression in patients with CVD."

METHODS

3a) Description of trial design (such as parallel, factorial) including allocation ratio

Does your paper address CONSORT subitem 3a? *

Copy and paste relevant sections from the manuscript (include quotes in quotation marks "like this" to indicate direct quotes from your manuscript), or elaborate on this item by providing additional information not in the ms, or briefly explain why the item is not applicable/relevant for your study

"Patients were randomised 1.1 to nine-week iCBT (intervention group) or online discussion forum (ODF) (active control group) generated by an independent statistician using Stata v.13 proc Ralloc with a block size of two."

3b) Important changes to methods after trial commencement (such as eligibility criteria), with reasons

Does your paper address CONSORT subitem 3b? *

Copy and paste relevant sections from the manuscript (include quotes in quotation marks "like this" to indicate direct quotes from your manuscript), or elaborate on this item by providing additional information not in the ms, or briefly explain why the item is not applicable/relevant for your study

"No changes were made to the program during the trial."

3b-i) Bug fixes, Downtimes, Content Changes

Bug fixes, Downtimes, Content Changes: ehealth systems are often dynamic systems. A description of changes to methods therefore also includes important changes made on the intervention or comparator during the trial (e.g., major bug fixes or changes in the functionality or content) (5-iii) and other “unexpected events” that may have influenced study design such as staff changes, system failures/downtimes, etc. [2].

subitem not at all important ○ ○ ◉ ○ ○ essential

Does your paper address subitem 3b-i?

Copy and paste relevant sections from the manuscript (include quotes in quotation marks "like this" to indicate direct quotes from your manuscript), or elaborate on this item by providing additional information not in the ms, or briefly explain why the item is not applicable/relevant for your study

"No changes were made to the program during the trial"

4a) Eligibility criteria for participants

Does your paper address CONSORT subitem 4a? *

Copy and paste relevant sections from the manuscript (include quotes in quotation marks "like this" to indicate direct quotes from your manuscript), or elaborate on this item by providing additional information not in the ms, or briefly explain why the item is not applicable/relevant for your study

"Patients were eligible for inclusion if they were 18 years or older and were receiving CVD treatment according to current guidelines for heart failure, coronary artery disease and atrial fibrillation from the European Society of Cardiology [15-17], had stable CVD (New York Heart Association (NYHA) class I-III), with no hospitalisation related to CVD in the past four weeks, and suffered at least mild depressive symptoms (Patient Health Questionnaire-9 (PHQ-9) score ≥5 [18]). Furthermore, patients needed to have regular access to a computer with an internet connection, access to a mobile phone, and be willing to participate in a treatment for their depression"

4a-i) Computer / Internet literacy

Computer / Internet literacy is often an implicit “de facto” eligibility criterion - this should be explicitly clarified.

subitem not at all important ○ ○ ○ ◉ ○ essential

Does your paper address subitem 4a-i?

Copy and paste relevant sections from the manuscript (include quotes in quotation marks "like this" to indicate direct quotes from your manuscript), or elaborate on this item by providing additional information not in the ms, or briefly explain why the item is not applicable/relevant for your study

To be eligible the particpants, " needed to have regular access to a computer with an internet connection, access to a mobile phone."

4a-ii) Open vs. closed, web-based vs. face-to-face assessments:

Open vs. closed, web-based vs. face-to-face assessments: Mention how participants were recruited (online vs. offline), e.g., from an open access website or from a clinic, and clarify if this was a purely web-based trial, or there were face-to-face components (as part of the intervention or for assessment), i.e., to what degree got the study team to know the participant. In online-only trials, clarify if participants were quasi-anonymous and whether having multiple identities was possible or whether technical or logistical measures (e.g., cookies, email confirmation, phone calls) were used to detect/prevent these.

subitem not at all important ○ ○ ○ ◉ ○ essential

Does your paper address subitem 4a-ii? *

Copy and paste relevant sections from the manuscript (include quotes in quotation marks "like this" to indicate direct quotes from your manuscript), or elaborate on this item by providing additional information not in the ms, or briefly explain why the item is not applicable/relevant for your study

"Patients who had registered on the study website were asked to complete a web-based screening form that collected data about depression as assessed by PHQ, demographics, smoking and alcohol habits, CVD diagnosis, time since diagnosis of CVD, NYHA classification, comorbidities and medications for CVD, depression, sleep problems and anxiety. Patients assessed as potential participants (i.e. had CVD and scored ≥5 on the PHQ-9) were contacted by telephone by study nurses, with clinical experience from psychiatric and cardiac care, to give information about the study, to answer questions and to check any uncertainties in the screening form, as well as to assess severity of CVD and depression. During the telephone interview the Mini International Neuropsychiatric Interview (MINI) version 5 panel A (i.e. depression) and panel C (i.e. suicidal ideation) were used to establish presence of at least mild depression and severity (i.e. presence of core symptoms or not, depression severity, and suicidal ideation)."

"Self-reported data were collected via a set of questionnaires that were completed through study website."

4a-iii) Information giving during recruitment

Information given during recruitment. Specify how participants were briefed for recruitment and in the informed consent procedures (e.g., publish the informed consent documentation as appendix, see also item X26), as this information may have an effect on user self-selection, user expectation and may also bias results.

subitem not at all important ○ ○ ○ ◉ ○ essential

Does your paper address subitem 4a-iii?

Copy and paste relevant sections from the manuscript (include quotes in quotation marks "like this" to indicate direct quotes from your manuscript), or elaborate on this item by providing additional information not in the ms, or briefly explain why the item is not applicable/relevant for your study

"Patients assessed as potential participants (i.e. had CVD and scored ≥5 on the PHQ-9) were contacted by telephone by study nurses, with clinical experience from psychiatric and cardiac care, to give information about the study", to answer questions and to check any uncertainties in the screening form, as well as to assess severity of CVD and depression. During the telephone interview the Mini International Neuropsychiatric Interview (MINI) version 5 panel A (i.e. depression) and panel C (i.e. suicidal ideation) were used to establish presence of at least mild depression and severity (i.e. presence of core symptoms or not, depression severity, and suicidal ideation). Those who fulfilled the inclusion and exclusion criteria were included in the study."

"All included participants signed a written informed consent."

4b) Settings and locations where the data were collected

Does your paper address CONSORT subitem 4b? *

Copy and paste relevant sections from the manuscript (include quotes in quotation marks "like this" to indicate direct quotes from your manuscript), or elaborate on this item by providing additional information not in the ms, or briefly explain why the item is not applicable/relevant for your study

"Self-reported data were collected via a set of questionnaires that were completed through study website."

4b-i) Report if outcomes were (self-)assessed through online questionnaires

Clearly report if outcomes were (self-)assessed through online questionnaires (as common in web-based trials) or otherwise.

subitem not at all important ○ ○ ○ ◉ ○ essential

Does your paper address subitem 4b-i? *

Copy and paste relevant sections from the manuscript (include quotes in quotation marks "like this" to indicate direct quotes from your manuscript), or elaborate on this item by providing additional information not in the ms, or briefly explain why the item is not applicable/relevant for your study

"Self-reported data were collected via a set of questionnaires that were completed through study website."

4b-ii) Report how institutional affiliations are displayed

Report how institutional affiliations are displayed to potential participants [on ehealth media], as affiliations with prestigious hospitals or universities may affect volunteer rates, use, and reactions with regards to an intervention.(Not a required item – describe only if this may bias results)

subitem not at all important ○ ○ ◉ ○ ○ essential

Does your paper address subitem 4b-ii?

Copy and paste relevant sections from the manuscript (include quotes in quotation marks "like this" to indicate direct quotes from your manuscript), or elaborate on this item by providing additional information not in the ms, or briefly explain why the item is not applicable/relevant for your study

This is not adressed in the manuscript. However, this information is provided on the study home-site. This information was also provided in the invitation letters.

5) The interventions for each group with sufficient details to allow replication, including how and when they were actually administered

5-i) Mention names, credential, affiliations of the developers, sponsors, and owners

Mention names, credential, affiliations of the developers, sponsors, and owners [6] (if authors/evaluators are owners or developer of the software, this needs to be declared in a “Conflict of interest” section or mentioned elsewhere in the manuscript).

subitem not at all important ○ ◉ ○ ○ ○ essential

Does your paper address subitem 5-i?

Copy and paste relevant sections from the manuscript (include quotes in quotation marks "like this" to indicate direct quotes from your manuscript), or elaborate on this item by providing additional information not in the ms, or briefly explain why the item is not applicable/relevant for your study

This question is not applicable for our study. Our iCBT program is not a commercial product.

5-ii) Describe the history/development process

Describe the history/development process of the application and previous formative evaluations (e.g., focus groups, usability testing), as these will have an impact on adoption/use rates and help with interpreting results.

subitem not at all important ○ ○ ◉ ○ ○ essential

Does your paper address subitem 5-ii?

Copy and paste relevant sections from the manuscript (include quotes in quotation marks "like this" to indicate direct quotes from your manuscript), or elaborate on this item by providing additional information not in the ms, or briefly explain why the item is not applicable/relevant for your study

"The intervention group participated in a nine-week iCBT programme that was initially tailored to fit patients with heart failure and depression and who have undergone pilot testing [19, 20]."

5-iii) Revisions and updating

Revisions and updating. Clearly mention the date and/or version number of the application/intervention (and comparator, if applicable) evaluated, or describe whether the intervention underwent major changes during the evaluation process, or whether the development and/or content was “frozen” during the trial. Describe dynamic components such as news feeds or changing content which may have an impact on the replicability of the intervention (for unexpected events see item 3b).

subitem not at all important ○ ○ ◉ ○ ○ essential

Does your paper address subitem 5-iii?

Copy and paste relevant sections from the manuscript (include quotes in quotation marks "like this" to indicate direct quotes from your manuscript), or elaborate on this item by providing additional information not in the ms, or briefly explain why the item is not applicable/relevant for your study

"No changes were made to the program during the trial"

5-iv) Quality assurance methods

Provide information on quality assurance methods to ensure accuracy and quality of information provided [1], if applicable.

subitem not at all important ○ ◉ ○ ○ ○ essential

Does your paper address subitem 5-iv?

Copy and paste relevant sections from the manuscript (include quotes in quotation marks "like this" to indicate direct quotes from your manuscript), or elaborate on this item by providing additional information not in the ms, or briefly explain why the item is not applicable/relevant for your study

we describe that all patients had a diagnosis of "CVD according to ICD codes Invitations were sent by post to all patients with at least one diagnosis of atrial fibrillation or atrial flutter (International Classification of Diseases (ICD)codes I48. or I49.), coronary heart disease (ICD codes I20. or I25.), and heart failure (ICD codes I42. or I50."

"Masking of patients was not possible since the intervention is a cognitive behavioural intervention. It was not necessary to mask outcome measures because these were automatically collected via the study platform."

5-v) Ensure replicability by publishing the source code, and/or providing screenshots/screen-c

Ensure replicability by publishing the source code, and/or providing screenshots/screen-capture video, and/or providing flowcharts of the algorithms used. Replicability (i.e., other researchers should in principle be able to replicate the study) is a hallmark of scientific reporting.

subitem not at all important ○ ○ ○ ◉ ○ essential

Does your paper address subitem 5-v?

Copy and paste relevant sections from the manuscript (include quotes in quotation marks "like this" to indicate direct quotes from your manuscript), or elaborate on this item by providing additional information not in the ms, or briefly explain why the item is not applicable/relevant for your study

Screenshots and flowcharts are included in the manuscript

5-vi) Digital preservation

Digital preservation: Provide the URL of the application, but as the intervention is likely to change or disappear over the course of the years; also make sure the intervention is archived (Internet Archive, webcitation.org, and/or publishing the source code or screenshots/videos alongside the article). As pages behind login screens cannot be archived, consider creating demo pages which are accessible without login.

subitem not at all important ○ ◉ ○ ○ ○ essential

Does your paper address subitem 5-vi?

Copy and paste relevant sections from the manuscript (include quotes in quotation marks "like this" to indicate direct quotes from your manuscript), or elaborate on this item by providing additional information not in the ms, or briefly explain why the item is not applicable/relevant for your study

To some extent not applicable for our study. Our iCBT program is not a regular treatment for CVD patients with depression. If implementation of the ICBT program would come into question, this program would be located in the official Swedish Medical Webbforum 1177

5-vii) Access

Access: Describe how participants accessed the application, in what setting/context, if they had to pay (or were paid) or not, whether they had to be a member of specific group. If known, describe how participants obtained “access to the platform and Internet” [1]. To ensure access for editors/reviewers/readers, consider to provide a “backdoor” login account or demo mode for reviewers/readers to explore the application (also important for archiving purposes, see vi).

subitem not at all important ○ ○ ◉ ○ ○ essential

Does your paper address subitem 5-vii? *

Copy and paste relevant sections from the manuscript (include quotes in quotation marks "like this" to indicate direct quotes from your manuscript), or elaborate on this item by providing additional information not in the ms, or briefly explain why the item is not applicable/relevant for your study

"Invitations were sent by post to all patients with at least one diagnosis of atrial fibrillation or atrial flutter (International Classification of Diseases (ICD)codes I48. or I49.), coronary heart disease (ICD codes I20. or I25.), and heart failure (ICD codes I42. or I50.) and at least one outpatient visits or hospitalisation during the last year before recruitment."

"Patients interested in study participation were instructed to register on the study website (www.dohart.se) (see screenshot in Multimedia appendix 1)"

5-viii) Mode of delivery, features/functionalities/components of the intervention and compara

Describe mode of delivery, features/functionalities/components of the intervention and comparator, and the theoretical framework [6] used to design them (instructional strategy [1], behaviour change techniques, persuasive features, etc., see e.g., [7, 8] for terminology). This includes an in-depth description of the content (including where it is coming from and who developed it) [1],” whether [and how] it is tailored to individual circumstances and allows users to track their progress and receive feedback” [6]. This also includes a description of communication delivery channels and – if computer-mediated communication is a component – whether communication was synchronous or asynchronous [6]. It also includes information on presentation strategies [1], including page design principles, average amount of text on pages, presence of hyperlinks to other resources, etc. [1].

subitem not at all important ○ ○ ○ ○ ◉ essential

Does your paper address subitem 5-viii? *

Copy and paste relevant sections from the manuscript (include quotes in quotation marks "like this" to indicate direct quotes from your manuscript), or elaborate on this item by providing additional information not in the ms, or briefly explain why the item is not applicable/relevant for your study

"The intervention group participated in a nine-week iCBT programme that was initially tailored to fit patients with heart failure and depression and who have undergone pilot testing [19, 20]. The programme consisted of seven modules including goal setting, psychoeducation, problem solving, behavioural activation and a summary module. For the present study, the programme was further developed to fit a broader group of cardiac patients by adding psycho-educative modules about coronary artery disease, atrial fibrillation and atrial flutter. Each module consisted of text, short videos and weekly homework assignments. Screenshots of different modules and homework assignments can be found in Multimedia appendix 2."

"Written feedback was provided on each assignment in the end of each week by three nurses"

"Participants also had the opportunity to ask questions about the feedback or the content of the module using a message function in the study platform. These questions were answered within 24 hours during business days."

"As recommended in a systematic review[13] we used an active control group that participated in a web-based moderated discussion forum (i.e. ODF group) where new discussion topics were presented each week over a nine-week period. The topic was introduced without any extended background in a presentation consisting of statements and questions. The discussion was performed in writing. One of the members in the study group (i.e. GM) was responsible for monitoring the ODF. After nine weeks the participants in the ODF were offered the iCBT programme."

5-ix) Describe use parameters

Describe use parameters (e.g., intended “doses” and optimal timing for use). Clarify what instructions or recommendations were given to the user, e.g., regarding timing, frequency, heaviness of use, if any, or was the intervention used ad libitum.

subitem not at all important ○ ○ ○ ◉ ○ essential

Does your paper address subitem 5-ix?

Copy and paste relevant sections from the manuscript (include quotes in quotation marks "like this" to indicate direct quotes from your manuscript), or elaborate on this item by providing additional information not in the ms, or briefly explain why the item is not applicable/relevant for your study

"a nine-week iCBT programme that was initially tailored to fit patients with heart failure and depression and who have undergone pilot testing [19, 20]"

"Each module consisted of text, short videos and weekly homework assignments." Screenshots of different modules and homework assignments can be found in Multimedia appendix 2."

"Written feedback was provided on each assignment in the end of each week by three nurses"

"Participants who did not complete the homework assignments received a maximum of 3 written reminders during a consecutive period of 2 weeks."

5-x) Clarify the level of human involvement

Clarify the level of human involvement (care providers or health professionals, also technical assistance) in the e-intervention or as co-intervention (detail number and expertise of professionals involved, if any, as well as “type of assistance offered, the timing and frequency of the support, how it is initiated, and the medium by which the assistance is delivered”. It may be necessary to distinguish between the level of human involvement required for the trial, and the level of human involvement required for a routine application outside of a RCT setting (discuss under item 21 – generalizability).

subitem not at all important ○ ○ ○ ◉ ○ essential

Does your paper address subitem 5-x?

Copy and paste relevant sections from the manuscript (include quotes in quotation marks "like this" to indicate direct quotes from your manuscript), or elaborate on this item by providing additional information not in the ms, or briefly explain why the item is not applicable/relevant for your study

"Written feedback was provided on each assignment in the end of each week by three nurses"

"Participants also had the opportunity to ask questions about the feedback or the content of the module using a message function in the study platform. These questions were answered within 24 hours during business days."

5-xi) Report any prompts/reminders used

Report any prompts/reminders used: Clarify if there were prompts (letters, emails, phone calls, SMS) to use the application, what triggered them, frequency etc. It may be necessary to distinguish between the level of prompts/reminders required for the trial, and the level of prompts/reminders for a routine application outside of a RCT setting (discuss under item 21 – generalizability).

subitem not at all important ○ ○ ○ ◉ ○ essential

Does your paper address subitem 5-xi? *

Copy and paste relevant sections from the manuscript (include quotes in quotation marks "like this" to indicate direct quotes from your manuscript), or elaborate on this item by providing additional information not in the ms, or briefly explain why the item is not applicable/relevant for your study

"Participants who did not complete the homework assignments received a maximum of 3 written reminders during a consecutive period of 2 weeks."

5-xii) Describe any co-interventions (incl. training/support)

Describe any co-interventions (incl. training/support): Clearly state any interventions that are provided in addition to the targeted eHealth intervention, as ehealth intervention may not be designed as stand-alone intervention. This includes training sessions and support [1]. It may be necessary to distinguish between the level of training required for the trial, and the level of training for a routine application outside of a RCT setting (discuss under item 21 – generalizability.

subitem not at all important (●) ( ) ( ) ( ) ( ) essential

Does your paper address subitem 5-xii? *

Copy and paste relevant sections from the manuscript (include quotes in quotation marks "like this" to indicate direct quotes from your manuscript), or elaborate on this item by providing additional information not in the ms, or briefly explain why the item is not applicable/relevant for your study

Not apllicable. This study did not include any co-internvention

6a) Completely defined pre-specified primary and secondary outcome measures, including how and when they were assessed

Does your paper address CONSORT subitem 6a? *

Copy and paste relevant sections from the manuscript (include quotes in quotation marks "like this" to indicate direct quotes from your manuscript), or elaborate on this item by providing additional information not in the ms, or briefly explain why the item is not applicable/relevant for your study

"Self-reported data were collected via a set of questionnaires that were completed through study website"

"Primary outcome
PHQ-9 was used to measure level of depression at baseline and at nine weeks' follow-up"

"Secondary outcomes
MADRS-S [21] was used as a security measurement for depressive symptoms and suicidal thoughts during the intervention."

"Health Related Quality of Life (HRQoL) was measured by Short Form 12 (SF-12) [27] and EQ-VAS [28]. The SF-12 measures HRQoL via 12 items selected from the Short Form-36 [27]. "

"Adherence was determined based on number of completed modules (iCBT group) and number of activities in the ODF (ODF group), which was provided by the study platform."

6a-i) Online questionnaires: describe if they were validated for online use and apply CHERRIES

If outcomes were obtained through online questionnaires, describe if they were validated for online use and apply CHERRIES items to describe how the questionnaires were designed/deployed [9].

subitem not at all important ( ) ( ) (●) ( ) ( ) essential

Does your paper address subitem 6a-i?

Copy and paste relevant sections from manuscript text

"The PHQ-9 has been found to be reliable and valid for detecting depression [18, 22] and also in patients with CVD (i.e. heart failure) [23]. The PHQ-9 has also been found valid in the computer format [24]."

"MADRS-S has been found to be a valid and reliable tool when administered by the internet [26]."

6a-ii) Describe whether and how “use” (including intensity of use/dosage) was defined/measu

Describe whether and how “use” (including intensity of use/dosage) was defined/measured/monitored (logins, logfile analysis, etc.). Use/adoption metrics are important process outcomes that should be reported in any ehealth trial.

subitem not at all important ○ ○ ◉ ○ ○ essential

Does your paper address subitem 6a-ii?

Copy and paste relevant sections from manuscript text

"Adherence was determined based on number of completed modules (iCBT group) and number of activities in the ODF (ODF group), which was provided by the study platform."

6a-iii) Describe whether, how, and when qualitative feedback from participants was obtained

Describe whether, how, and when qualitative feedback from participants was obtained (e.g., through emails, feedback forms, interviews, focus groups).

subitem not at all important ○ ○ ◉ ○ ○ essential

Does your paper address subitem 6a-iii?

Copy and paste relevant sections from manuscript text

"Participants also had the opportunity to ask questions about the feedback or the content of the module using a message function in the study platform. These questions were answered within 24 hours during business days."

6b) Any changes to trial outcomes after the trial commenced, with reasons

Does your paper address CONSORT subitem 6b? *

Copy and paste relevant sections from the manuscript (include quotes in quotation marks "like this" to indicate direct quotes from your manuscript), or elaborate on this item by providing additional information not in the ms, or briefly explain why the item is not applicable/relevant for your study

"No changes were made to the program during the trial."

7a) How sample size was determined

NPT: When applicable, details of whether and how the clustering by care provides or centers was addressed

7a-i) Describe whether and how expected attrition was taken into account when calculating th

Describe whether and how expected attrition was taken into account when calculating the sample size.

subitem not at all important ○ ○ ○ ◉ ○ essential

Does your paper address subitem 7a-i?

Copy and paste relevant sections from manuscript title (include quotes in quotation marks "like this" to indicate direct quotes from your manuscript), or elaborate on this item by providing additional information not in the ms, or briefly explain why the item is not applicable/relevant for your study

"Power calculation based on effect size in meta-analysis on iCBT [30] showed that a total of 122 participants would be needed to detect at least a moderate effect size on depression (effect size=0·5, alpha=0·05 (Z=1.96), power 0·80 (Z −0.84)). Due to expected drop-outs, the size of the study sample was set to 140 patients."

7b) When applicable, explanation of any interim analyses and stopping guidelines

Does your paper address CONSORT subitem 7b? *

Copy and paste relevant sections from the manuscript (include quotes in quotation marks "like this" to indicate direct quotes from your manuscript), or elaborate on this item by providing additional information not in the ms, or briefly explain why the item is not applicable/relevant for your study

Not applicable for this study

8a) Method used to generate the random allocation sequence

NPT: When applicable, how care providers were allocated to each trial group

Does your paper address CONSORT subitem 8a? *

Copy and paste relevant sections from the manuscript (include quotes in quotation marks "like this" to indicate direct quotes from your manuscript), or elaborate on this item by providing additional information not in the ms, or briefly explain why the item is not applicable/relevant for your study

"The randomisation was performed by a study team member (GM) who was blinded to screening and baseline data. None of the telephone interviewers had access to the random sequence. Patients were randomised 1.1 to nine-week iCBT (intervention group) or online discussion forum (ODF) (active control group) generated by an independent statistician using Stata v.13 proc Ralloc with a block size of two."

8b) Type of randomisation; details of any restriction (such as blocking and block size)

Does your paper address CONSORT subitem 8b? *

Copy and paste relevant sections from the manuscript (include quotes in quotation marks "like this" to indicate direct quotes from your manuscript), or elaborate on this item by providing additional information not in the ms, or briefly explain why the item is not applicable/relevant for your study

"Patients were randomised 1.1 to nine-week iCBT (intervention group) or online discussion forum (ODF) (active control group) generated by an independent statistician using Stata v.13 proc Ralloc with a block size of two."

9) Mechanism used to implement the random allocation sequence (such as sequentially numbered containers), describing any steps taken to conceal the sequence until interventions were assigned

Does your paper address CONSORT subitem 9? *

Copy and paste relevant sections from the manuscript (include quotes in quotation marks "like this" to indicate direct quotes from your manuscript), or elaborate on this item by providing additional information not in the ms, or briefly explain why the item is not applicable/relevant for your study

"The randomisation was performed by a study team member (GM) who was blinded to screening and baseline data. None of the telephone interviewers had access to the random sequence.

10) Who generated the random allocation sequence, who enrolled participants, and who assigned participants to interventions

Does your paper address CONSORT subitem 10? *

Copy and paste relevant sections from the manuscript (include quotes in quotation marks "like this" to indicate direct quotes from your manuscript), or elaborate on this item by providing additional information not in the ms, or briefly explain why the item is not applicable/relevant for your study

"The randomisation was performed by a study team member (GM) who was blinded to screening and baseline data."

11a) If done, who was blinded after assignment to interventions (for example, participants, care providers, those assessing outcomes) and how

NPT: Whether or not administering co-interventions were blinded to group assignment

11a-i) Specify who was blinded, and who wasn’t

Specify who was blinded, and who wasn’t. Usually, in web-based trials it is not possible to blind the participants [1, 3] (this should be clearly acknowledged), but it may be possible to blind outcome assessors, those doing data analysis or those administering co-interventions (if any).

subitem not at all important ○ ○ ○ ◉ ○ essential

Does your paper address subitem 11a-i? *

Copy and paste relevant sections from the manuscript (include quotes in quotation marks "like this" to indicate direct quotes from your manuscript), or elaborate on this item by providing additional information not in the ms, or briefly explain why the item is not applicable/relevant for your study

"Masking of patients was not possible since the intervention is a cognitive behavioural intervention. It was not necessary to mask outcome measures because these were automatically collected via the study platform."

11a-ii) Discuss e.g., whether participants knew which intervention was the “intervention of inte

Informed consent procedures (4a-ii) can create biases and certain expectations - discuss e.g., whether participants knew which intervention was the “intervention of interest” and which one was the “comparator”.

subitem not at all important ○ ○ ○ ◉ ○ essential

Does your paper address subitem 11a-ii?

Copy and paste relevant sections from the manuscript (include quotes in quotation marks "like this" to indicate direct quotes from your manuscript), or elaborate on this item by providing additional information not in the ms, or briefly explain why the item is not applicable/relevant for your study

"Masking of patients was not possible since the intervention is a cognitive behavioural intervention."

11b) If relevant, description of the similarity of interventions

(this item is usually not relevant for ehealth trials as it refers to similarity of a placebo or sham intervention to a active medication/intervention)

Does your paper address CONSORT subitem 11b? *

Copy and paste relevant sections from the manuscript (include quotes in quotation marks "like this" to indicate direct quotes from your manuscript), or elaborate on this item by providing additional information not in the ms, or briefly explain why the item is not applicable/relevant for your study

Ditt svar

12a) Statistical methods used to compare groups for primary and secondary outcomes

NPT: When applicable, details of whether and how the clustering by care providers or centers was addressed

Does your paper address CONSORT subitem 12a? *

Copy and paste relevant sections from the manuscript (include quotes in quotation marks "like this" to indicate direct quotes from your manuscript), or elaborate on this item by providing additional information not in the ms, or briefly explain why the item is not applicable/relevant for your study

"we used an active control group that participated in a web-based moderated discussion forum (i.e. ODF group) where new discussion topics were presented each week over a nine-week period. The topic was introduced without any extended background in a presentation consisting of statements and questions. The discussion was performed in writing. One of the members in the study group (i.e. GM) was responsible for monitoring the ODF. After nine weeks the participants in the ODF were offered the iCBT programme. This information was provided in writing on the study homepage and orally during the telephone interview."

12a-i) Imputation techniques to deal with attrition / missing values

Imputation techniques to deal with attrition / missing values: Not all participants will use the intervention/comparator as intended and attrition is typically high in ehealth trials. Specify how participants who did not use the application or dropped out from the trial were treated in the statistical analysis (a complete case analysis is strongly discouraged, and simple imputation techniques such as LOCF may also be problematic [4]).

subitem not at all important ○ ○ ○ ◉ ○ essential

Does your paper address subitem 12a-i? *

Copy and paste relevant sections from the manuscript (include quotes in quotation marks "like this" to indicate direct quotes from your manuscript), or elaborate on this item by providing additional information not in the ms, or briefly explain why the item is not applicable/relevant for your study

"We followed the recommendations from the European Medicines Agency for statistical analysis [31] and used analysis of covariance (ANCOVA) which allows adjusting for baseline scores and regression to the mean [32]. Missing data in the ANCOVA was imputed using last observation carried forward (LOCF) since no consensus on how to best pool f-statistics was available [33]. However, LOCF has received criticism [34] we therefore also applied mixed models analysis with multiple imputed data as a sensitivity analysis. A total of 40 imputations were performed using the outcome variables and variables from baseline characteristics that had a correlation greater than $r \geq 0.5$ with the outcome variables as predictors [35]. Multiple imputed data sets as well as raw data on primary outcome are available in Multimedia appendix 3."

12b) Methods for additional analyses, such as subgroup analyses and adjusted analyses

Does your paper address CONSORT subitem 12b? *

Copy and paste relevant sections from the manuscript (include quotes in quotation marks "like this" to indicate direct quotes from your manuscript), or elaborate on this item by providing additional information not in the ms, or briefly explain why the item is not applicable/relevant for your study

"Per-protocol analysis was performed to evaluate categorical improvements of depression as measured by: minimally clinical important change defined by a decrease of 5 points or more on the PHQ-9 [36]; the proportion of non-depressed (i.e. PHQ-9 score <5) at 9 weeks follow-up; and the proportion of non- or mildly depressed (i.e. PHQ-9 score <10) at 9 weeks' follow-up. Based on these analyses, the numbers needed to treat (NNT) were calculated. We also calculated NNT for MADRS-S as a categorical variable (no depression = MADRS-S score 0-12, or depression score ≥13). Per-protocol analysis was also performed to analyse and compare the change in level of depression in relation to the number of CBT modules and the number of activities in the ODF completed (i.e. adherence to the programme)."

X26) REB/IRB Approval and Ethical Considerations [recommended as subheading under "Methods"] (not a CONSORT item)

X26-i) Comment on ethics committee approval

subitem not at all important ○ ○ ○ ○ ◉ essential

Does your paper address subitem X26-i?

Copy and paste relevant sections from the manuscript (include quotes in quotation marks "like this" to indicate direct quotes from your manuscript), or elaborate on this item by providing additional information not in the ms, or briefly explain why the item is not applicable/relevant for your study

"The study was approved by the regional ethical review board in Linköping Sweden (no. 2016/72-31)"

x26-ii) Outline informed consent procedures

Outline informed consent procedures e.g., if consent was obtained offline or online (how? Checkbox, etc.?), and what information was provided (see 4a-ii). See [6] for some items to be included in informed consent documents.

subitem not at all important ○ ○ ○ ○ ○ essential

Does your paper address subitem X26-ii?

Copy and paste relevant sections from the manuscript (include quotes in quotation marks "like this" to indicate direct quotes from your manuscript), or elaborate on this item by providing additional information not in the ms, or briefly explain why the item is not applicable/relevant for your study

"All included participants signed a written informed consent."

X26-iii) Safety and security procedures

Safety and security procedures, incl. privacy considerations, and any steps taken to reduce the likelihood or detection of harm (e.g., education and training, availability of a hotline)

subitem not at all important ○ ○ ○ ○ ○ essential

Does your paper address subitem X26-iii?

Copy and paste relevant sections from the manuscript (include quotes in quotation marks "like this" to indicate direct quotes from your manuscript), or elaborate on this item by providing additional information not in the ms, or briefly explain why the item is not applicable/relevant for your study

"For safety issues and as requested by the ethical review board, we screened suicidality and worsening in depressive symptoms by weekly screening using the Montgomery Åsberg Depression Rating Scale-self rating (MADRS-S) [21]. Patients who scored five or higher on MADRS-S item 9 (zest for life) were contacted by the research team and if necessary, advised to seek acute psychiatric care."

RESULTS

13a) For each group, the numbers of participants who were randomly assigned, received intended treatment, and were analysed for the primary outcome

NPT: The number of care providers or centers performing the intervention in each group and the number of patients treated by each care provider in each center

Does your paper address CONSORT subitem 13a? *

Copy and paste relevant sections from the manuscript (include quotes in quotation marks "like this" to indicate direct quotes from your manuscript), or elaborate on this item by providing additional information not in the ms, or briefly explain why the item is not applicable/relevant for your study

Described in figure 1.

"Out of these, 144 were included and randomised to either iCBT (n=72) or ODF (n=72). Main reason for exclusion was a screening score <5 on PHQ-9 (i.e. 20 %, n=56), did not-complete the screening form (i.e. 9 %, n=20) or declined depression during the telephone interview (i.e. 6 %, n=16) (Figure 1). The number of patients who did not complete the nine-week trial period was similar in the two groups (iCBT n=7 [10 %]; ODF n=10 [14 %])."

13b) For each group, losses and exclusions after randomisation, together with reasons

Does your paper address CONSORT subitem 13b? (NOTE: Preferably, this is shown in a CONS

Copy and paste relevant sections from the manuscript (include quotes in quotation marks "like this" to indicate direct quotes from your manuscript), or elaborate on this item by providing additional information not in the ms, or briefly explain why the item is not applicable/relevant for your study

Described in figure 1.

"Out of these, 144 were included and randomised to either iCBT (n=72) or ODF (n=72). Main reason for exclusion was a screening score <5 on PHQ-9 (i.e. 20 %, n=56), did not-complete the screening form (i.e. 9 %, n=20) or declined depression during the telephone interview (i.e. 6 %, n=16) (Figure 1). The number of patients who did not complete the nine-week trial period was similar in the two groups (iCBT n=7 [10 %]; ODF n=10 [14 %])."

13b-i) Attrition diagram

Strongly recommended: An attrition diagram (e.g., proportion of participants still logging in or using the intervention/comparator in each group plotted over time, similar to a survival curve) or other figures or tables demonstrating usage/dose/engagement.

subitem not at all important ○ ○ ○ ○ ○ essential

Does your paper address subitem 13b-i?

Copy and paste relevant sections from the manuscript or cite the figure number if applicable (include quotes in quotation marks "like this" to indicate direct quotes from your manuscript), or elaborate on this item by providing additional information not in the ms, or briefly explain why the item is not applicable/relevant for your study

Described in figure 1.

14a) Dates defining the periods of recruitment and follow-up

Does your paper address CONSORT subitem 14a? *

Copy and paste relevant sections from the manuscript (include quotes in quotation marks "like this" to indicate direct quotes from your manuscript), or elaborate on this item by providing additional information not in the ms, or briefly explain why the item is not applicable/relevant for your study

"Recruitment took place between January 2017 and February 2018"

14a-i) Indicate if critical “secular events” fell into the study period

Indicate if critical “secular events” fell into the study period, e.g., significant changes in Internet resources available or “changes in computer hardware or Internet delivery resources”

subitem not at all important ◉ ○ ○ ○ ○ essential

Does your paper address subitem 14a-i?

Copy and paste relevant sections from the manuscript (include quotes in quotation marks "like this" to indicate direct quotes from your manuscript), or elaborate on this item by providing additional information not in the ms, or briefly explain why the item is not applicable/relevant for your study

No secular events fell into the internvention period.

14b) Why the trial ended or was stopped (early)

Does your paper address CONSORT subitem 14b? *

Copy and paste relevant sections from the manuscript (include quotes in quotation marks "like this" to indicate direct quotes from your manuscript), or elaborate on this item by providing additional information not in the ms, or briefly explain why the item is not applicable/relevant for your study

Not applicable

15) A table showing baseline demographic and clinical characteristics for each group

NPT: When applicable, a description of care providers (case volume, qualification, expertise, etc.) and centers (volume) in each group

Does your paper address CONSORT subitem 15? *

Copy and paste relevant sections from the manuscript (include quotes in quotation marks "like this" to indicate direct quotes from your manuscript), or elaborate on this item by providing additional information not in the ms, or briefly explain why the item is not applicable/relevant for your study

Table 1

15-i) Report demographics associated with digital divide issues

In ehealth trials it is particularly important to report demographics associated with digital divide issues, such as age, education, gender, social-economic status, computer/Internet/ehealth literacy of the participants, if known.

subitem not at all important ○ ○ ○ ○ ○ essential

Does your paper address subitem 15-i? *

Copy and paste relevant sections from the manuscript (include quotes in quotation marks "like this" to indicate direct quotes from your manuscript), or elaborate on this item by providing additional information not in the ms, or briefly explain why the item is not applicable/relevant for your study

Table 1

"Baseline characteristics (Table 1) were similar between the two groups. The mean age was 63 years (SD 12) and 38 % (n=55) were women and 37 % (n=53) had college/university level of education. With regard to cardiac diagnosis, 56 % (n=81) had atrial fibrillation/flutter, 44% (n=65) had coronary heart disease and 26 % (n=38) had heart failure. More than one quarter of the total population had two or more cardiac diagnoses (i.e. 28 %, n=40). In addition, 53 % (n=76) had hypertension, 15 % (n=21) diabetes and 13 % (n=19) had a previous stroke/transitory ischemic attack. Regarding pharmacological treatment for CVD, 76 % (n=110) were on betablockers, 48 % (n=67) were on Renin Angiotensin Aldosterone System blocking agents, and 89 % (n=110) used antiplatelets/anticoagulantia. Mean score on PHQ-9 was 10.47 (SD 4.78) and 14% (n=20) were prescribed antidepressant treatment."

16) For each group, number of participants (denominator) included in each analysis and whether the analysis was by original assigned groups

16-i) Report multiple “denominators” and provide definitions

Report multiple “denominators” and provide definitions: Report N’s (and effect sizes) “across a range of study participation [and use] thresholds” [1], e.g., N exposed, N consented, N used more than x times, N used more than y weeks, N participants “used” the intervention/comparator at specific pre-defined time points of interest (in absolute and relative numbers per group). Always clearly define “use” of the intervention.

subitem not at all important ○ ○ ○ ◉ ○ essential

Does your paper address subitem 16-i? *

Copy and paste relevant sections from the manuscript (include quotes in quotation marks "like this" to indicate direct quotes from your manuscript), or elaborate on this item by providing additional information not in the ms, or briefly explain why the item is not applicable/relevant for your study

"Intention to treat analysis (iCBT n=72 and ODF n=72) of the primary outcome of depression as measured by PHQ-9 at 9-weeks"

" In the per-protocol analysis (iCBT n=65 and ODF n=62) aimed at comparing categorical improvements in depression at 9 weeks’ follow-up, the proportion of patients who had a clinically important improvement in depression (i.e. decrease with ≥5 points in PHQ-9) was significantly larger in the iCBT group than the ODF (43% (n=28) vs. 24% (n=15), P=.024). There was also a significant larger proportion of non-depressed (PHQ-9<5) (35% (n=23) vs 21% (n=13), P=.028) or mildly/non-depressed (PHQ-9<10) (82% (n=53) vs 66% (n=41), P=.049) (Figure 3) in the iCBT group compared to the ODF group."

16-ii) Primary analysis should be intent-to-treat

Primary analysis should be intent-to-treat, secondary analyses could include comparing only “users”, with the appropriate caveats that this is no longer a randomized sample (see 18-i).

subitem not at all important ○ ○ ○ ○ ◉ essential

Does your paper address subitem 16-ii?

Copy and paste relevant sections from the manuscript (include quotes in quotation marks "like this" to indicate direct quotes from your manuscript), or elaborate on this item by providing additional information not in the ms, or briefly explain why the item is not applicable/relevant for your study

"Intention to treat analysis (iCBT n=72 and ODF n=72) of the primary outcome of depression as measured by PHQ-9 at 9-weeks"

17a) For each primary and secondary outcome, results for each group, and the estimated effect size and its precision (such as 95% confidence interval)

Does your paper address CONSORT subitem 17a? *

Copy and paste relevant sections from the manuscript (include quotes in quotation marks "like this" to indicate direct quotes from your manuscript), or elaborate on this item by providing additional information not in the ms, or briefly explain why the item is not applicable/relevant for your study

"Intention to treat analysis (iCBT n=72 and ODF n=72) of the primary outcome of depression as measured by PHQ-9 at 9-weeks' follow-up showed a statistically significant moderate treatment effect of iCBT (mean group difference -2.34 [95 % CI -3.58 to -1.10], P <.001., Cohens d=0.62) compared to ODF

Table 2

17a-i) Presentation of process outcomes such as metrics of use and intensity of use

In addition to primary/secondary (clinical) outcomes, the presentation of process outcomes such as metrics of use and intensity of use (dose, exposure) and their operational definitions is critical. This does not only refer to metrics of attrition (13-b) (often a binary variable), but also to more continuous exposure metrics such as "average session length". These must be accompanied by a technical description how a metric like a "session" is defined (e.g., timeout after idle time) [1] (report under item 6a).

subitem not at all important ○ ○ ◉ ○ ○ essential

Does your paper address subitem 17a-i?

Copy and paste relevant sections from the manuscript (include quotes in quotation marks "like this" to indicate direct quotes from your manuscript), or elaborate on this item by providing additional information not in the ms, or briefly explain why the item is not applicable/relevant for your study

"With regard to adherence, a total of 60% (n=43) in the iCBT group completed all seven modules, whereas 82% (n=59) completed more than half of the modules (i.e. four or more). In the ODF group 27% (n=20) of the patients completed nine or more activities (e.g. reading or posting) in the forum threads. In the per-protocol analysis performed to compare the change in level of depression relation to adherence (Multiemedia appendix 3, Tables A2 and A3), we first compared those who had completed at least one iCBT treatment module (n=69) to those with at least one activity in the ODF (n=49), in which a significant and moderate effect of iCBT was found (P<.001., Cohens d=0.69) In the next step, those in the iCBT group who had completed all seven modules (n=43) were compared to those in the ODF who had a least nine activities (n=20), a significant and large effect of iCBT (P=.002, Cohens d=0.89) was found."

17b) For binary outcomes, presentation of both absolute and relative effect sizes is recommended

Does your paper address CONSORT subitem 17b? *

Copy and paste relevant sections from the manuscript (include quotes in quotation marks "like this" to indicate direct quotes from your manuscript), or elaborate on this item by providing additional information not in the ms, or briefly explain why the item is not applicable/relevant for your study

Not applicable

18) Results of any other analyses performed, including subgroup analyses and adjusted analyses, distinguishing pre-specified from exploratory

Does your paper address CONSORT subitem 18? *

Copy and paste relevant sections from the manuscript (include quotes in quotation marks "like this" to indicate direct quotes from your manuscript), or elaborate on this item by providing additional information not in the ms, or briefly explain why the item is not applicable/relevant for your study

" In the per-protocol analysis (iCBT n=65 and ODF n=62) aimed at comparing categorical improvements in depression at 9 weeks' follow-up, the proportion of patients who had a clinically important improvement in depression (i.e. decrease with ≥5 points in PHQ-9) was significantly larger in the iCBT group than the ODF (43% (n=28) vs. 24% (n=15), P=.024). There was also a significant larger proportion of non-depressed (PHQ-9<5) (35% (n=23) vs 21% (n=13), P=.028) or mildly/non-depressed (PHQ-9<10) (82% (n=53) vs 66% (n=41), P=.049) (Figure 3) in the iCBT group compared to the ODF group."

18-i) Subgroup analysis of comparing only users

A subgroup analysis of comparing only users is not uncommon in ehealth trials, but if done, it must be stressed that this is a self-selected sample and no longer an unbiased sample from a randomized trial (see 16-iii).

subitem not at all important ○ ○ ◉ ○ ○ essential

Does your paper address subitem 18-i?

Copy and paste relevant sections from the manuscript (include quotes in quotation marks "like this" to indicate direct quotes from your manuscript), or elaborate on this item by providing additional information not in the ms, or briefly explain why the item is not applicable/relevant for your study

"With regard to adherence, a total of 60% (n=43) in the iCBT group completed all seven modules, whereas 82% (n=59) completed more than half of the modules (i.e. four or more). In the ODF group 27% (n=20) of the patients completed nine or more activities (e.g. reading or posting) in the forum threads. In the per-protocol analysis performed to compare the change in level of depression relation to adherence (Multiemedia appendix 3, Tables A2 and A3), we first compared those who had completed at least one iCBT treatment module (n=69) to those with at least one activity in the ODF (n=49), in which a significant and moderate effect of iCBT was found (P<.001., Cohens d=0.69) In the next step, those in the iCBT group who had completed all seven modules (n=43) were compared to those in the ODF who had a least nine activities (n=20), a significant and large effect of iCBT (P=.002, Cohens d=0.89) was found."

19) All important harms or unintended effects in each group

(for specific guidance see CONSORT for harms)

Does your paper address CONSORT subitem 19? *

Copy and paste relevant sections from the manuscript (include quotes in quotation marks "like this" to indicate direct quotes from your manuscript), or elaborate on this item by providing additional information not in the ms, or briefly explain why the item is not applicable/relevant for your study

"Regarding safety, one patient in the iCBT group and three patients in the ODF group demonstrated an increase of more than five points on PHQ-9 when comparing individual baseline and post measures. At baseline, three patients in each group reported a score of two or more on item nine in PHQ-9 (thought of being better off dead). At nine weeks, these numbers had decreased to one and two in iCBT and ODF respectively. On two occasions, one patient in the iCBT group scored above four on MADRS-S item nine (zest for life) during the predefined weekly safety measures. The corresponding numbers for the ODF group were three occasions among three patients. These patients were contacted by telephone for an evaluation, but no patient was discontinued from the study due to high risk of suicide or deterioration in depression."

19-i) Include privacy breaches, technical problems

Include privacy breaches, technical problems. This does not only include physical “harm” to participants, but also incidents such as perceived or real privacy breaches [1], technical problems, and other unexpected/unintended incidents. “Unintended effects” also includes unintended positive effects [2].

subitem not at all important (●) ( ) ( ) ( ) ( ) essential

Does your paper address subitem 19-i?

Copy and paste relevant sections from the manuscript (include quotes in quotation marks "like this" to indicate direct quotes from your manuscript), or elaborate on this item by providing additional information not in the ms, or briefly explain why the item is not applicable/relevant for your study

Non reported during the intervention

19-ii) Include qualitative feedback from participants or observations from staff/researchers

Include qualitative feedback from participants or observations from staff/researchers, if available, on strengths and shortcomings of the application, especially if they point to unintended/unexpected effects or uses. This includes (if available) reasons for why people did or did not use the application as intended by the developers.

subitem not at all important (●) ( ) ( ) ( ) ( ) essential

Does your paper address subitem 19-ii?

Copy and paste relevant sections from the manuscript (include quotes in quotation marks "like this" to indicate direct quotes from your manuscript), or elaborate on this item by providing additional information not in the ms, or briefly explain why the item is not applicable/relevant for your study

Not reported in the present manuscript. We have qualiative studies ongoing where this information will be presented.

DISCUSSION

22) Interpretation consistent with results, balancing benefits and harms, and considering other relevant evidence

NPT: In addition, take into account the choice of the comparator, lack of or partial blinding, and unequal expertise of care providers or centers in each group

22-i) Restate study questions and summarize the answers suggested by the data, starting witl

Restate study questions and summarize the answers suggested by the data, starting with primary outcomes and process outcomes (use).

subitem not at all important ( ) ( ) ( ) (●) ( ) essential

Does your paper address subitem 22-i? *

Copy and paste relevant sections from the manuscript (include quotes in quotation marks "like this" to indicate direct quotes from your manuscript), or elaborate on this item by providing additional information not in the ms, or briefly explain why the item is not applicable/relevant for your study

"To our knowledge, this is the first adequately powered randomised controlled trial aimed at evaluating the effect of a nurse-delivered iCBT programme for depression in patients diagnosed with CVD. We found that the programme, which was tailored to fit the context of cardiovascular disease, was more effective than the online discussion forum to reduce depression and improve HRQoL."

22-ii) Highlight unanswered new questions, suggest future research
Highlight unanswered new questions, suggest future research.

subitem not at all important ○ ○ ○ ◉ ○ essential

Does your paper address subitem 22-ii?
Copy and paste relevant sections from the manuscript (include quotes in quotation marks "like this" to indicate direct quotes from your manuscript), or elaborate on this item by providing additional information not in the ms, or briefly explain why the item is not applicable/relevant for your study

"An alternative tailored iCBT programme could be designed to incorporate a collaboration between CVD nurses, psychologists and cardiologists, who could all respond on-demand, depending on the needs of the patients."

20) Trial limitations, addressing sources of potential bias, imprecision, and, if relevant, multiplicity of analyses

20-i) Typical limitations in ehealth trials
Typical limitations in ehealth trials: Participants in ehealth trials are rarely blinded. Ehealth trials often look at a multiplicity of outcomes, increasing risk for a Type I error. Discuss biases due to non-use of the intervention/usability issues, biases through informed consent procedures, unexpected events.

subitem not at all important ○ ○ ◉ ○ ○ essential

Does your paper address subitem 20-i? *
Copy and paste relevant sections from the manuscript (include quotes in quotation marks "like this" to indicate direct quotes from your manuscript), or elaborate on this item by providing additional information not in the ms, or briefly explain why the item is not applicable/relevant for your study

"Another limitation is that feedback was delivered by three nurses from the study team, thus it is possible that they were biased by being too ambitious in their feedback. However, feedback on homework assignments was only provided once a week and the time for feedback for those who had completed all seven modules was 13 minutes per week and patient. Moreover, the therapeutic part in iCBT is imbedded within the text whereas the feedback focusses to encouragement, and confirming and reflecting upon the patients' homework assignments, preparing for the next module, and when required discussing psycho-educative CVD related issues (e.g. sexuality, treatment side effects and symptoms)[9]."

21) Generalisability (external validity, applicability) of the trial findings

NPT: External validity of the trial findings according to the intervention, comparators, patients, and care providers or centers involved in the trial

21-i) Generalizability to other populations
Generalizability to other populations: In particular, discuss generalizability to a general Internet population, outside of a RCT setting, and general patient population, including applicability of the study results for other organizations

subitem not at all important ○ ○ ◉ ○ ○ essential

Does your paper address subitem 21-i?

Copy and paste relevant sections from the manuscript (include quotes in quotation marks "like this" to indicate direct quotes from your manuscript), or elaborate on this item by providing additional information not in the ms, or briefly explain why the item is not applicable/relevant for your study

"this study has shown that a nurse-delivered and tailored iCBT programme decreased depression and improved HRQoL in CVD patients with depression."

21-ii) Discuss if there were elements in the RCT that would be different in a routine applicatior

Discuss if there were elements in the RCT that would be different in a routine application setting (e.g., prompts/reminders, more human involvement, training sessions or other co-interventions) and what impact the omission of these elements could have on use, adoption, or outcomes if the intervention is applied outside of a RCT setting.

subitem not at all important ○ ○ ◉ ○ ○ essential

Does your paper address subitem 21-ii?

Copy and paste relevant sections from the manuscript (include quotes in quotation marks "like this" to indicate direct quotes from your manuscript), or elaborate on this item by providing additional information not in the ms, or briefly explain why the item is not applicable/relevant for your study

"An alternative tailored iCBT programme could be designed to incorporate a collaboration between CVD nurses, psychologists and cardiologists, who could all respond on-demand, depending on the needs of the patients."

OTHER INFORMATION

23) Registration number and name of trial registry

Does your paper address CONSORT subitem 23? *

Copy and paste relevant sections from the manuscript (include quotes in quotation marks "like this" to indicate direct quotes from your manuscript), or elaborate on this item by providing additional information not in the ms, or briefly explain why the item is not applicable/relevant for your study

"and is registered at clinicaltrials.gov (NCT02778074)."

24) Where the full trial protocol can be accessed, if available

Does your paper address CONSORT subitem 24? *

Cite a Multimedia Appendix, other reference, or copy and paste relevant sections from the manuscript (include quotes in quotation marks "like this" to indicate direct quotes from your manuscript), or elaborate on this item by providing additional information not in the ms, or briefly explain why the item is not applicable/relevant for your study

T"he study protocol can be found at the project homepage (https://liu.se/forskning/dohart)"

25) Sources of funding and other support (such as supply of drugs), role of funders

Does your paper address CONSORT subitem 25? *

Copy and paste relevant sections from the manuscript (include quotes in quotation marks "like this" to indicate direct quotes from your manuscript), or elaborate on this item by providing additional information not in the ms, or briefly explain why the item is not applicable/relevant for your study

"Role of funding source
The funders of the study had no role in the study design, data collection, analysis, interpretation or writing of the manuscript. PJ, JL, and MW had full access to all study data. All authors had final responsibility for the decision to submit for publication."

X27) Conflicts of Interest (not a CONSORT item)

X27-i) State the relation of the study team towards the system being evaluated

In addition to the usual declaration of interests (financial or otherwise), also state the relation of the study team towards the system being evaluated, i.e., state if the authors/evaluators are distinct from or identical with the developers/sponsors of the intervention.

subitem not at all important ○ ○ ◉ ○ ○ essential

Does your paper address subitem X27-i?

Copy and paste relevant sections from the manuscript (include quotes in quotation marks "like this" to indicate direct quotes from your manuscript), or elaborate on this item by providing additional information not in the ms, or briefly explain why the item is not applicable/relevant for your study

"Declaration of interest
None of the authors declare any competing interest."

About the CONSORT EHEALTH checklist

As a result of using this checklist, did you make changes in your manuscript? *

○ yes, major changes

◉ yes, minor changes

○ no

What were the most important changes you made as a result of using this checklist?

We added the information where the study protocol could be found.

How much time did you spend on going through the checklist INCLUDING making changes in

4 hours

As a result of using this checklist, do you think your manuscript has improved? *

◉ yes

○ no

○ Övrigt:

Would you like to become involved in the CONSORT EHEALTH group?

This would involve for example becoming involved in participating in a workshop and writing an "Explanation and Elaboration" document

○ yes

◉ no

○ Övrigt:

Any other comments or questions on CONSORT EHEALTH

Ditt svar

STOP - Save this form as PDF before you click submit

To generate a record that you filled in this form, we recommend to generate a PDF of this page (on a Mac, simply select "print" and then select "print as PDF") before you submit it.

When you submit your (revised) paper to JMIR, please upload the PDF as supplementary file.

Don't worry if some text in the textboxes is cut off, as we still have the complete information in our database. Thank you!

Final step: Click submit !

Click submit so we have your answers in our database!

SKICKA

Skicka aldrig lösenord med Google Formulär

Det här innehållet har varken skapats eller godkänts av Google. Anmäl otillåten användning - Användarvillkor

Google Formulär
